# Study protocol: families and childhood transitions study (FACTS) – a longitudinal investigation of the role of the family environment in brain development and risk for mental health disorders in community based children

**DOI:** 10.1186/s12887-017-0905-x

**Published:** 2017-06-30

**Authors:** J.G. Simmons, O.S. Schwartz, K. Bray, C. Deane, E. Pozzi, S. Richmond, J. Smith, N. Vijayakumar, M.L. Byrne, M.L. Seal, M.B.H. Yap, N.B. Allen, S.L. Whittle

**Affiliations:** 1 Melbourne School of Psychological Sciences, The University of Melbourne, Parkville, Australia; 2 Melbourne Neuropsychiatry Centre, Department of Psychiatry, The University of Melbourne and Melbourne Health, Parkville, Australia; 3 Department of Psychology, The University of Oregon, Eugene, OR USA; 4 Developmental Imaging, MRI Department, Royal Children’s Hospital, Parkville, Australia; 5 Department of Paediatrics, The University of Melbourne, Parkville, Australia; 6 School of Psychological Sciences, Monash Institute of Cognitive and Clinical Neurosciences, Monash University, Clayton, Australia; 7 Melbourne School of Population and Global Health, The University of Melbourne, Parkville, Australia; 8 Murdoch Childrens Research Institute, Parkville, Australia

**Keywords:** Brain development, Late childhood, Parenting, Social disadvantage, Mental health, Hormones, Adrenarche, Protocol, MRI

## Abstract

**Background:**

Extant research has demonstrated that parenting behaviour can be a significant contributor to the development of brain structure and mental health during adolescence. Nonetheless, there is limited research examining these relationships during late childhood, and particularly in the critical period of brain development occurring between 8 and 10 years of age. The effects of the family environment on the brain during late childhood may have significant implications for later functioning, and particularly mental health. The Families and Childhood Transitions Study (FACTS) is a multidisciplinary longitudinal cohort study of brain development and mental health, with two waves of data collection currently funded, occurring 18-months apart, when child participants are aged approximately 8- and 10-years old.

**Methods/design:**

Participants are 163 children (*M* age *[SD]* = 8.44 [0.34] years, 76 males) and their mothers (*M* age *[SD]* = 40.34 [5.43] years). Of the 163 families who consented to participate, 156 completed a video-recorded and observer-coded dyadic interaction task and 153 completed a child magnetic resonance imaging brain scan at baseline. Families were recruited from lower socioeconomic status (SES) areas to maximise rates of social disadvantage and variation in parenting behaviours. All experimental measures and tasks completed at baseline are repeated at an 18-month follow-up, excluding the observer coded family interaction tasks. The baseline assessment was completed in October 2015, and the 18-month follow up will be completed May 2017.

**Discussion:**

This study, by examining the neurobiological and mental health consequences of variations in parenting, has the potential to significantly advance our understanding of child development and risk processes. Recruitment of lower SES families will also allow assessment of resilience factors given the poorer outcomes often associated with this population.

**Electronic supplementary material:**

The online version of this article (doi:10.1186/s12887-017-0905-x) contains supplementary material, which is available to authorized users.

## Background

Research from our group has demonstrated that parenting behaviour can be a significant contributor to the development of brain structure, as well as to psychological adjustment during adolescence [1–9]. However, these results, and the broader literature (e.g., [10–12]), suggest that the effects of parenting behaviours on brain development may be equally, if not more important, earlier in life. The influence of parenting is likely to be especially pronounced during the period of late childhood (i.e., 8 to 10 years), as this phase of development marks the first stages of a wave of significant brain growth and reorganization, second only to infancy in terms of its extent and significance for functional development (see [13]). These neurodevelopmental processes mean that the brain is highly plastic, and hence potentially more sensitive to environmental influence in comparison to other periods of life. Thus, the effects of the family environment on the brain during late childhood may have significant implications for later functioning. These effects may be particularly important to investigate in the context of social disadvantage, given that the stressors associated with disadvantage may lead to sub-optimal parenting behaviours and other domestic stressors for children [14], and given the evidence that parenting behaviours are a critical mediator between social disadvantage and poor child outcomes [15].

This paper is a methodological description of the *Families and Childhood Transitions Study* (FACTS). This longitudinal study aims to examine the influence of the family environment, and particularly parenting and stressful events, on child brain development and mental health during late childhood.

### Impact of parenting on brain development

The effect of parenting on children’s development has long been the subject of empirical study. Our group and others have provided substantial evidence that children and adolescents are at risk for poorer psychosocial and mental health outcomes as a result of exposure to adverse family environments characterized by elevated levels of harsh parenting and conflictual interactions between parent and child [9, 16–24]. In particular, we have provided evidence that emotionally aggressive and dysphoric parenting behaviours observed in laboratory tasks prospectively predict adverse outcomes in adolescence [3, 5, 25–27].

There are two key principles in understanding how and why parenting influences brain development. Firstly, *brain plasticity* refers to the collection of mechanisms involved in the organization and reorganization of the brain and its connections throughout the lifespan. Secondly, *sensitive periods* refer to temporal windows during which environmental factors can influence neurobiological systems in a more acute and/or persistent way. A general principle is that sensitive periods are associated with increased environmental influence due to increased plasticity [28]. During these times, maximal reorganization of synapses (formation followed by pruning) permits experiential processes to guide neural configuration, in either helpful or harmful ways [29].

Much of the research to date has focused on the influence of very early, or very severe family environmental factors (e.g., maltreatment) on the brain. A focus on very early factors is important given that the brain is undergoing a period of maximal growth prenatally and during the first years of life [30]. Numerous studies, most involving animals (but some in humans), have documented the negative effects of early postnatal exposure to stress and social deprivation on both brain and behavioural development, and on long-term outcomes. For example, rodent studies have shown that significant disruption to maternal care is associated with enduring systemic physiological changes in the functioning of the hypothalamic-pituitary-adrenal (HPA) axis [31–33], which plays a critical role in development, stress responsivity and affective functioning. The majority of human studies have investigated the effects of relatively extreme adverse family environments on the brain. The structure and function of the hippocampus, amygdala and prefrontal cortex appear to be most implicated [34, 35], which is consistent with their role in the activity of the HPA axis [36, 37]. For example, adult and adolescent studies consistently report reductions in hippocampal volume in the context of maltreatment histories [34, 38]. However, a meta-analysis has provided evidence of significantly *larger* hippocampal volume in *children* with maltreatment-related PTSD compared to controls, with such enlargement further associated with greater externalizing behaviours [39]. This contrast between adult and child findings suggests developmental effects in the influence of trauma on the brain.

Parenting practices in the more ‘normative’ range, in addition to influencing children’s cognitive, social and emotional development, also likely influence children’s neurobiological development. In a previous multi-method prospective study, we investigated associations between measures of positive and negative affective parenting behaviours during parent-child interactions and measures of brain structure during adolescence (i.e., namely, volumes of subcortical and prefrontal regions known to be critical for emotional/behavioural reactivity and regulation) [7, 24]; and see [40] for an overview). In 2011, Belsky and Haan [41] published an influential review calling for further research to build on the evidence base provided by our work. Since then, we have undertaken further research examining the impact of parenting behaviours on brain structure longitudinally [1, 2, 42]. This topic has become a growing research area, with more groups internationally seeking to replicate and extend our findings (e.g., [12, 43–46]. Most of this research has found both negative (e.g., hostility [43], aggression [24]) and positive (e.g., praise and encouragement [46]) parenting behaviours to be associated with the structure of brain regions involved in stress and emotion regulation, and executive functioning. Further, alterations in neurobiological development have also been found to mediate the relationship between parenting and other developmental outcomes.

### Impact of social disadvantage and parenting on child/adolescent development

Social disadvantage is associated with an increase in family exposure to negative life events and stressors, such as family and community violence, family dissolution, changing abode, unemployment, and job uncertainty [47]. The family’s response to such stressors is one of the most significantly cited mediators in the impact of social disadvantage on a child’s cognitive and socio-emotional development [15, 48]. In particular, these stressors may generate psychological distress in parents such that they become less able to provide their children with adequate responsive and supportive caregiving, and are more likely to adopt punitive, coercive parenting styles [15]. Studies consistently report associations between social disadvantage and inadequate parenting, such as reduced warmth and involvement [49], inadequate supervision [50], and harsh or inconsistent discipline [49, 51].

Whilst social disadvantage is associated with poorer parenting practices, this is not the case for all families, and there is evidence that in conditions of social disadvantage, the maintenance of positive parenting practices could represent a protective factor by providing a buffer, or reducing the negative impact on children’s development [52, 53]. For example, Brody and colleagues [54] found that children experiencing social adversity *and* supportive and involved parenting had more favourable outcomes (e.g., better self-regulation and lower symptoms of depression and aggression) than children without supportive parenting.

In this study, we have selected participants from communities experiencing higher levels of social disadvantage. This is for two reasons. First, well-established social gradients in family dysfunction mean that studying such a group provides a methodological advantage by increasing the variance in parenting characteristics within the sample, thus providing more experimental power. The second and more compelling reason is that the high prevalence of family dysfunction and poor child outcomes within these communities renders them a more likely setting for targeted prevention and early intervention efforts that will ultimately be informed by this work. As such, conducting the investigation amongst families of higher social disadvantage provides the study with greater external validity.

We will investigate both negative and positive aspects of parenting in order to address how parenting behaviour may contribute to both risk *and* resilience. Further, because of evidence both that adverse parenting environments influence endocrine function [55, 56] and that the latter has the potential to influence brain development [57], we will also investigate the mediating role of endocrine function in the link between parenting behaviour and child brain development.

### Aims

This project aims to establish whether aversive (i.e., aggressive and dysphoric) parenting influences childhood brain development in the context of social disadvantage. We also aim to investigate whether positive parenting practices might buffer or protect children against the deleterious effect of social disadvantage on brain development. Finally, we propose to investigate whether HPA axis function mediates the relationship between measures of parenting and brain development, and whether other biological markers (such as genetics and immune function) mediate and/or moderate associations. To address these aims, we will conduct a comprehensive assessment of parenting and other aspects of the family environment, with a key focus being on observed indices of parenting behaviour. Two waves of brain imaging will be conducted, with a focus on assessment of neuroanatomical changes in three key brain regions – the hippocampus, amygdala and prefrontal cortex (PFC). Additionally, we will conduct a comprehensive assessment of the HPA axis, including the influence of relevant genetic variation and endocrine function at both time points, which will comprise measurement of basal salivary and hair cortisol, DHEA-S, DHEA, and testosterone. Finally, we will also examine the relationships with immune function, via the measurement of salivary C-reactive protein (CRP), secretory immunoglobulin-A (SIgA) and other relevant markers. This project will provide an innovative and critical knowledge base, allowing us to more fully understand the pathways by which social disadvantage and family environmental factors influence outcomes across the lifespan.

### Specific aims

1. *Assess the influence of adverse and positive parenting, in the context of social disadvantage, on the development of child brain structure during the neurobiologically sensitive developmental period of late childhood using two waves of imaging data (*i.e.*, assessments at ages 8 and 10).*
2. *Assess if and how HPA axis short-term (salivary) and long-term (hair) basal activity mediates observed associations between parenting behaviours and late childhood brain development.*
3. *Assess if and how genetic and immune markers mediate and moderate observed associations between parenting behaviours and late childhood brain development.*
4. *Assess if and how the environmental and biological factors measured are associated with child mental health.*

## Methods/design

### Overall study design

FACTS is a multidisciplinary longitudinal cohort study of brain development, with two waves of data collection currently funded, occurring 18-months apart, when child participants are aged approximately 8- and 10-years old. Families were recruited from lower socioeconomic status areas, as detailed below, to maximise rates of social disadvantage and variation in parenting behaviours. All experimental measures completed at baseline are repeated at the follow-up, excluding the observer coded family interaction tasks. Additional measures are included at the follow-up. The baseline assessment was completed in October 2015, and the 18-month follow-up will be completed May 2017. Funding was obtained from the Australian Research Council (ARC; DP130103551). FACTS is based in both the Melbourne School of Psychological Sciences and the Melbourne Neuropsychiatry Centre at The University of Melbourne, Australia, with all MRI scans being carried out at the Royal Children’s Hospital (RCH), Parkville. Ethics approval was granted by the University of Melbourne Human Research Ethics Office (#1339904). The study adhered to the 'strengthening the reporting of observational studies in epidemiology' (STROBE; www.strobe-statement.org) guidelines. See Additional file 1 for STROBE cohort study ﻿checklist.

Further funding will be sought to enable the current investigation to follow up children and their families during adolescence, the period of peak onset for mental health disorders. This will permit further examination of the longitudinal and prospective relationships of parenting and family environment with brain development and functional and health outcomes.

### Recruitment

Participant recruitment commenced in September 2013. Recruitment was restricted to Melbourne metropolitan areas classified by the Australian Bureau of Statistics as falling within the lower tertile of socioeconomic disadvantage from the 2011 national Australian population census, compulsory for all residents [58]. Metropolitan areas were selected to facilitate follow up assessments and reduce participant travel burden. Multiple methods of recruitment were employed within selected areas to maximise participant numbers, and included:

- Recruitment booths at shopping centres
- Flyers and brochures in community centres
- Advertisements in school newsletters
- Recruitment through primary schools, with letters sent to parents with children in target age range. In the letter, families were asked to return a reply-paid form indicating whether they did, or did not want further information about FACTS. When this letter was sent back (with either response) the child was sent a small brain-shaped toy.

An ‘opt-in’ model of participation was used with all methods. To opt-in, the primary caregiver provided contact details and expressed interest in learning more about the study. The parents of interested families were contacted by telephone and provided more detailed information. A participant information and consent form (PICF) was then sent to families by post or email, and followed up with a phone call approximately two weeks later. Verbal consent was then obtained from child and parent participants, a screening questionnaire completed to assess inclusion/exclusion criteria (see Table 1), and experimental sessions scheduled. Parental participation was restricted to mothers as our prior studies suggested we would be unlikely to be able to recruit enough father-child dyads or alternate caregiver-child dyads within budgetary restraints (e.g., only 17–18% of parent participants who were not mothers [24]) to statistically compare the effects of these different types of relationships. These relationships are important areas for future research.

**Table 1:** Eligibility criteria for FACTS

Inclusion Criteria	Exclusion Criteria
Family lives within area coded as falling within the lower tertile of socioeconomic advantage in the State of Victoria;	History of head trauma or loss of consciousness;
Child aged between 8.0 and 9.25 years at the time of their participation;	History of clinically significant developmental or intellectual disorder;
Written consent provided by parent for their own participation;	Indications of claustrophobia;
Written consent provided by the parent and the child for the child’s participation; and,	Presence or likelihood of internal or external non-removable ferrous metals;
Verbal assent provided by the child.	Inability or unwillingness of participant or parent/guardian to provide informed consent.

### Participants

Participants comprised 163 children (*M* age *[SD]* = 8.44 (0.34) years, *n* males [%] = 76 [46.63]) and their mothers (*M* age *[SD]* = 40.34 [5.43] years). Of the 163 families who consented to participate, 153 completed an MRI scan at baseline and 156 completed the family interaction task. One family did not complete the interaction task as instructed and could not be scored, leaving usable interaction data for 155 children. A total of 609 families expressed interest in the study, however 320 declined to participate and a further 126 were excluded based upon eligibility criteria (see Table 1).

### Data collection procedures

#### Baseline assessment

Participating families were scheduled to attend two assessments. Assessments comprised: 1) the family interaction task (FIT) session; and, 2) the child brain MRI scan session at The RCH. All measures and tasks administered at both time points are summarised in Table 2, with further details provided in Additional file 2. The assessments were completed either on one day (*N* = 129, 79%), or across two days—with the majority of those completed within 3 weeks of each other (*N* = 27, 79%). The FIT assessment included the collection of child questionnaires, anthropometry and hair samples. The MRI session assessment included collection of IQ and handedness measures. The mother was provided with a questionnaire pack with all parent questionnaires at the first assessment — to be completed across assessments. The ordering of sessions varied according to MRI availability, however the majority were ordered with the FIT assessment first (*N* = 99, 61%).

**Table 2:** Summary information on measures collected at baseline and 18-month follow up

Measure Name [Reference]	Administered		Report on	Domain
	Baseline	18-Mnths		
*Tasks/Direct Measures*				
MRI brain scan - structural	✓	✓	C	Neurobiology
MRI brain scan – functional [67]	-	✓	C	Neurobiology
Family Interaction Task (mother and child) [1, 3]	✓	-	M/C	Parenting / Child Behaviour
Anthropometry	✓	✓	C	Physical Development
Saliva samples	✓	✓	C	Neurobiology
Hair samples	✓	✓	C	Neurobiology
Wechsler Intelligence Scale for Children (WISC-IV) [78]	✓	✓	C	IQ
Silent films task [79]	-	✓	C	Social Cognition
*Child Questionnaires*				
Attachment Q. for Children (AQC) [99]	✓	✓	C	Attachment
Brief Multidimensional Student’s Life Satisfaction Scale (B-MSLSS) [100]	-	✓	C	Wellbeing
Children’s Depression Inventory (CDI-2) [101]	✓	✓	C	Symptoms
Children’s Rejection Sensitivity Q. (CRSQ) [102]	✓	✓	C	Symptoms / Behaviour
Edinburgh Handedness Inventory (EHI) [103]	✓	✓	C	Handedness
Empathy Q. (EQ) [104], [105]	-	✓	C	Social Cognition
Kern’s Security Scale (KSS) [106]	✓	✓	C	Attachment
*Parent Questionnaires*				
Alabama Parenting Q. (APQ) [107]	✓	✓	M	Parenting
Adult Rejection Sensitivity Q. (ARSQ) [108]	✓	✓	M	Symptoms/Behaviour
Alcohol, Smoking and Substance Involvement Screening Test (ASSIST) [109]	✓	✓	M	Substance Use
Beck Anxiety Inventory (BAI) [110]	✓	✓	M	Symptoms
Composite Abuse Scale (CAS) [111]	✓	✓	M	Abuse – Family Environ.
Child Behaviour Checklist (CBCL) [112]	✓	✓	C	Symptoms
Conflict Behaviour Q. (CBQ) [113]	✓	✓	M/C	Family Environment
Coping with Children’s Negative Emotions Scale (CCNES) [114]	✓	✓	M	Parenting
Children’s Depression Inventory – Parent (CDI-2-P) [115]	✓	✓	C	Symptoms
Centre for Epidemiologic Studies Depression Scale (CESD) [116]	✓	✓	M	Symptoms
Child Health Q. (CHQ) [117]	✓	✓	C	Health/Wellbeing
Children’s Report of Parental Behaviour Inventory – Parent Report (CRPBI-PR) [118]	✓	✓	M	Parenting
Lifetime Incidence of Traumatic Events (LITE) [119]	✓	✓	C	Trauma/Abuse
Multidimensional Neglectful Behaviour Scale (MNBS) [120]	✓	✓	M	Abuse - Neglect
Parental Reactions to Children’s Positive Emotions Scale (PRCPS) [24]	✓	✓	M	Parenting
Pubertal Development Scale (PDS) [121]	✓	✓	C	Physical Development
Sexual Maturity Scale (SMS) [122]	✓	✓	C	Physical Development
*Parent Interviews*				
Demographics/Health [77]	✓	✓	M & F/C	Demographics/Parent Mental Health/SES/Child Stressful Events/ Exclusions
*Teacher Questionnaire*				
Social Skills Improvement Scale (SSIS) [59]	-	✓	C	Social Competence

During the telephone call to scheduled assessments, and again at the beginning of the first appointment, a review of study participation requirements, eligibility, and informed consent was carried out. Verbal consent on the telephone call was recorded, and written consent obtained at the first assessment. Families were advised that all their information is confidential, except where limited by law, and that information collected will not be fed back to them, except where clinically significant abnormalities were indicated. Signed consent from a parent/guardian and verbal assent from children was required.

Two weeks prior to the first visit, families were sent a link to a web-based video about MRI scans at the RCH (https://vimeo.com/royalchildrenshospital/review/48121175/4dc0c867ef), and saliva collection kits (including an instructional video). Families were asked to collect child saliva samples one morning prior and on the morning of the first scheduled assessment, and return them at this assessment (see Measures section for further information).

At the end of the final assessment, participants took part in a debriefing interview and were provided with an information sheet on family and mental health resources. Any incomplete questionnaires were sent home to be returned in reply-paid envelopes. Parents were informed that they would be contacted in approximately 14-months time to arrange the phase 2 appointments, to be scheduled 18-months after completion of the baseline assessment.

#### Eighteen-month follow up

The 18-month follow up assessment is similar to the baseline assessment, with the major exception that the FIT is not repeated and thus only one experimental assessment is required. Additional questionnaires (including one rated by children’s teachers and one assessing children’s self-reported quality of life) and a theory of mind (‘Silent Films’) task for children were added to the assessment (see Table 2). Participating families are again sent saliva collection kits, and asked to attend the experimental session at the RCH. This appointment comprises the collection of questionnaire data (parent and child), IQ measures, anthropomorphic measurements and hair samples, and the completion of the Silent Films task and MRI brain scan. The MRI session is similar to that carried out at baseline, with the only difference being that an fMRI affective faces task has been added (see Measures for further details). Teachers are contacted subsequent to this visit, as detailed below.

##### Teacher assessment

Consent is collected from parents to contact the child’s primary teacher, and collect information about the child’s social functioning in the school setting. Where consent is given, schools are contacted after the follow up family assessment and teachers asked if they will participate. Permission is also required from school principals. When permission is given, the name of the child is given to the teacher, and the teacher is emailed a link to an online survey (built through Survey Monkey™) of the social skills subscale of the Social Skills Improvement System - Teacher Report (SSIS; [59]).

### Measures

#### Family interaction task

The Family Interaction Task (FIT) included two 15-min interactions that mother-child dyads completed together – an Event Planning Interaction (EPI), then a Problem Solving Interaction (PSI). The ordering of tasks was fixed because of concern that negative affective states elicited by the PSI had the potential to persist into the positive, EPI, if the latter were conducted second [60]. Fixing the task order also serves to reduce between-subjects variance (related to order), given that this is a correlational study focused on individual differences rather than group differences. During the EPI, participants planned between one and three enjoyable activities, such as ‘taking a trip or vacation’. These activities were chosen from the Pleasant Events Checklist (PEC), a modified version of the Pleasant Event Schedule [61]. During the PSI, participants chose three conflict-eliciting issues from the Issues Checklist (IC), such as ‘talking back to parents’ [62]. The dyads then problem solved each issue in detail. These conversations were video recorded using a separate digital video camera and microphone for each participant.

Audio-visual material recorded during the family interaction tasks was coded using the Family Interaction Macro-coding System (FIMS [63]). FIMS is a global coding method [64] adapted from a system devised by Smetana and colleagues [65]. Coders viewed each video and then provided 5-point Likert scale ratings on 67 items representing various dimensions designed to assess parent, child and family behaviour. FIMS items are outlined in a coding manual grouped under sections targeting interaction style, conflict, affect, control, parental behaviours, collaborative problem solving, and general family measures [66]. Further details on FIMS coding and item inclusion are provided in Additional file 2.

#### MRI brain scan

The MRI assessment at baseline commenced with a run-through of the MRI procedure with a mock scan in a replica MRI. This procedure provided safety information, tips for staying still, and assessed the child’s capacity to undertake the real scan, including observed anxiety levels (see Additional file 2 for further details). Parents complete a standard RCH MRI safety checklist for their child (and themselves if opting to sit in the scanner room with the child during the MRI). An MRI technician verbally reviews the MRI safety checklist with parents and children just prior to undertaking the MRI scan, and children are asked to choose a cartoon or movie they would like to watch during the scans (excluding the fMRI sequences). Parents are invited to remain in the MRI room while scanning is carried out. Subsequently, children are positioned comfortably in a supine orientation with their head located in a head-RF coil that is electrically isolated. The participant views a screen, via an angled adjustable mirror, on which all visual stimuli or video are presented using a back-projection system attached to a computer. Children wear MR-compatible headphones to reduce MRI noise, to allow them to hear instructions and speak with the MRI technician, and to hear the audio of any cartoons or movies they watch. Children are provided with an “Emergency Stop” button, in order to indicate to research staff if at any stage during the scan they feel distress and want to cease the procedure. Children complete a T1-weighted MPRAGE structural sequence, followed by a resting fMRI sequence (eyes closed), and a diffusion weighted imaging sequence. In cases where technical error or movement requir a particular sequence be repeated, a case-by-case assessment is made by research staff in discussion with the parent, child and MRI technician. Scanning takes an average of 30 min.

##### MRI brain scan parameters

Neuroimaging data are acquired on the 3 T Siemens TIM Trio scanner (Siemens, Erlangen, Germany) at the Murdoch Childrens Research Institute (MCRI). Participants lay supine with their head supported in a 32-channel head coil.

*Structural Scan –* T1-weighted images are acquired with motion correction (MPRAGE MoCo, repetition time = 2530 msec; echo time1 = 1.74 msec, echo time2 = 3.6 msec, echo time3 = 5.46 msec, echo time4 = 7.32 msec; flip angle = 7°, field of view = 256 × 256 mm^2^), which produced 176 contiguous 1.0 mm thick slices (voxel dimensions = 1.0 mm^3^). Sequence duration 5:19 min.

*Resting fMRI* – A continuous functional gradient-recalled acquisition sequence is conducted at rest to acquire 154 whole-brain T2*-weighted echo-planar volumes (repetition time = 2400 ms, echo time = 35 ms, flip angle = 90°; field of view = 210 × 210 mm^2^, 38 interleaved slices, voxel size of 3.3mm^3^). Complex field maps are obtained in order to correct for distortion caused by magnetic field inhomogeneities. Total sequence duration 6.18 min.

*DWI* – Diffusion weighted images are acquired (50 directions, b = 3000 s/mm^2^, 5 × b0 reference image, repetition time = 8500 msec; echo time = 112 msec; slices = 58; voxels = 2.3 mm^3^). In addition, reversed phase encoding scans (“Blip Up/Blip Down”) with same voxel parameters are acquired to assist with correction of spatial and intensity distortion. Total sequence duration 8:00 min.

*Affective faces fMRI task* – Participants are administered (at the 18-month follow-up only) a modified version of the emotional face-matching task originally reported by Hariri et al. [67]. In this task participants must either match the gender of faces presented (face condition), or match shapes (control condition). During each 4 s “face trial”, participants are presented with a target face (centre top) and two probe faces (bottom left and right) and are instructed to match the probe of the same gender to the target by pressing a button either on the left or right. During each 4 s “shape trial” participants are presented with a target shape (centre top) and two probe shapes (bottom left and right) and are instructed to match the probe of the same shape to the target by pressing a button either on the left or right. Each block consists of six consecutive trials containing angry or fearful faces (face condition) or shapes (control condition). A total of three 24-s blocks of each emotional face condition (i.e. angry and fearful) and six 24-s blocks of the control condition (shapes) are presented interleaved in a pseudo-randomized order. A fixation cross lasting 10-s is interspersed between each block. The total task time is 7 min. For each trial, response accuracy and response latency (reaction time) is obtained. Prior to the scan, participants complete a short practice version of the task with different emotional faces (happy and angry). Parameters include 136 whole-brain T2*-weighted echo-planar images (repetition time = 3000 ms, echo time = 35 ms, flip angle = 85°) within a field of view of 216x216mm^2^, with a voxel size of 3mm^3^. Forty interleaved slices are acquired. Total sequence duration 6:42 min.

#### Saliva samples

Children, with the help of a parent/guardian, are asked to collect a saliva sample on the day of and day prior to their visit immediately after waking, and prior to the consumption of food or tooth brushing. This is collected via the passive drool of whole saliva using a straw into test tubes (all equipment provided). Families are given a stopwatch to allow them to record how long it takes the child to provide enough saliva to reach the marked 2.5 ml line on the tube. Samples are then frozen in family’s freezers in provided sealed containers, and subsequently transported in provided coolers packed in Techni-Ice™ on the day of their assessment. Families are asked to minimise the time the samples spend out of the freezer, and all samples are checked on receipt. Samples are then frozen at the MCRI in a − 30 °C freezer till assay. At time of assay, samples are defrosted and centrifuged, with the supernatant assayed for levels of testosterone, DHEA and DHEA-S, as hormonal markers of adrenarcheal development, and cortisol as an important corollary of HPA axis development. Remaining supernatant is stored in 1 ml aliquots (typically ×3) in a − 80 °C freezer for future assays when funding allows, including other hormones (e.g., oestradiol) and immune system biomarkers (e.g., CRP and SIgA). Salivary assays of each of these biomarkers are now well-accepted substitutes for measuring serum levels [68, 69], although there are methodological idiosyncrasies for each (e.g., DHEA-S, see [70]). Hormonal assays for the baseline assessment were conducted at the MCRI, using Salimetrics ELISA kits. Kits from the same lot numbers were used, as were in-house controls. The inter-assay coefficients of variation (CVs) for the baseline assessment were: DHEA = 11.76%; DHEA-S = 13.77%; testosterone = 10.47%; cortisol = 5.32%. The intra-assay CVs were: DHEA = 9.03%; DHEA-S = 7.82%; testosterone = 8.17%; cortisol = 3.47%.

Saliva samples will also be utilised for the analysis of genetic and epigenetic variation. After removal of the supernatant from centrifuged samples, the cellular pellet is re-suspended in sterile phosphate-buffered saline and frozen at −80 °C. DNA will be extracted from these samples using established techniques [71].

#### Hair samples

Hair samples are collected for the assay of long term hormone levels in children [72], primarily cortisol, DHEA and testosterone. A section of hair approximately 1cm^2^ surface area on the scalp is taken from the posterior vertex. Longer samples are tied with string and the scalp-end of the sample clearly marked, while shorter samples are stored untied in an envelope. Samples are kept in controlled conditions away from light and extreme temperatures. Hair grows at a rate of approximately 1 cm per month [73], therefore a section of hair that is 3 cm in length provides an indication of hormonal output over several months. The sample is taken from the posterior vertex of the scalp as it has the lowest coefficient of variation for hormonal levels compared with other areas of the scalp [74]. A maximum length of 3 cm of hair is analysed to reduce damage to the hair from washing and sun exposure [75]. Hair assays for the baseline assessment were conducted by Stratech Scientific and processed and assayed as described previously [76], using Salimetrics ELISA kits for cortisol, DHEA and testosterone. The intra-assay coefficient of variation (CV) for the baseline assessment was 5.1%, and inter-assay CV 5.8%.

#### Anthropometry

Height, weight and waist measurements are collected and processed as previously described [70]. In brief, two measurements are obtained for height, weight and waist circumference; however, a third measurement is obtained where the prior two are not within a specified range (0.5 cm for height, 0.1 kg for weight, 0.5 cm for waist). The mean value is used in any further calculations if two measurements are taken, and the median value is used if three measurements are obtained. Further details are provided in Additional file 2.

#### Parent interviews

##### Demographics and health information

Detailed demographic information is collected including parental age, language spoken at home, race, ethnicity, child adoption status, and country of birth for the maternal and paternal grandparents, mother, father, and child. Also collected is socioeconomic data, such as residential neighbourhood, parental education, occupation and annual household income. Information about family structure is collected including significant caregivers and siblings (both biological and non-biological) living inside as well as outside the home. A brief mental health history of the primary caregivers is taken using the maternal-reported Lifetime Diagnosis of Psychiatric Symptoms – a brief interview using the dedicated subsection of the Kiddie-Schedule for Affective Disorders and Schizophrenia-Present and Lifetime Version (K-SADS-PL [77]). During this interview, mothers are asked to recall whether they or other primary care givers have been diagnosed, or experienced symptoms relating to, the following presentations: depression, anxiety, mania/hypomania, schizophrenia, psychotic symptoms, conduct or antisocial disorders, and substance use. If mental health diagnosis/symptoms are endorsed, mothers are asked whether treatment was received and if so what type – counselling, medication, etc. Information pertaining to the physical health of the child and primary maternal figure is also gathered for the purpose of MRI safety exclusions. A more extensive medical history is taken for the child, for the purpose of eligibility and exclusions, which includes: chronic and recent illnesses, current and previous medications, developmental disorders and stressful events experienced 3 months prior to the assessment.

#### Questionnaires – Child, Parent & Teacher

All questionnaires across baseline and the 18-month follow up are summarised in Table 2, with more detailed information provided in Additional file 2.

#### Intelligence quotient tasks

Three Wechsler Intelligence Scale for Children – Version IV [78] (WISC-IV; Australian Language Adaptation edition) subtests are used, specifically matrix reasoning, vocabulary and symbol search, in order to give an estimate of full scale IQ. Norms are based on 851 children and adolescents, aged 6 years to 16 years and 11 months, who participated in the Australian Standardisation Project [78].

#### Silent films task

The Silent Films task was developed to assess cognitive empathy/theory of mind [79]. The task is explained to the child initially, and examples provided. Children are then shown video clips on an iPad, and asked to answer questions after each clip. The task is comprised of five short film clips (mean length of 25 s) from a silent film: the 1923 romantic comedy, *Safety Last!,* directed by Newmeyer and Taylor*.* The clips depict instances of deception, false belief, belief-desire reasoning, and misunderstanding. The task requires participants to use their understanding of others beliefs and desires to explain the behaviour of characters in the clips, in response to a series of questions presented after each clip. The use of silent film clips broadens the task’s applicability for use with different language groups and with children of low verbal ability. It has been validated in 8–13 year olds and has good psychometric properties [79]. Further details are provided in Additional file 2.

### Power calculation

The most important statistical analysis procedures in this study will comprise correlational (including regression) analyses. These analyses will be used to predict outcomes amongst the participants (*n* = 163), depending on distributional characteristics. This will result in adequate power (>0.80; *p* = 0.05) to detect effect sizes of *r* = 0.2. Even with significant attrition in the longitudinal analyses (e.g., 20%), the study design will retain adequate power to detect effect sizes of *r* = 0.22. Across studies, investigators have consistently achieved less than 10% attrition in longitudinal designs. Therefore, the proposed study should have more than adequate power to detect effects in the expected range.

### Data analysis

Measures of observed negative and positive maternal affective behaviour will be used as the main predictors of interest in analyses. Covariates will be employed (e.g., parental mental health symptoms, other aspects of the family environment, previous experience of abuse or trauma, pubertal stage) where appropriate.

Aim 1: For whole-brain structural MRI analysis, a longitudinal processing scheme implemented in FreeSurfer (http://surfer.nmr.mgh.harvard.edu/ [80, 81]) will be used to test the effects of maternal behaviour on the development of brain structure (e.g., volume, cortical thickness). This procedure incorporates the subject-wise correlation of longitudinal data into the processing stream to reduce the measurement noise and ensure non-biased analysis of changes in structural measures. For whole brain vertex-wise analyses, resulting maps representing longitudinal change will be used. For ROI data, multilevel modelling [82] will be used to examine the effects of parental behaviour on structural brain development. This kind of modelling also provides consistent estimates when longitudinal data are unbalanced, due to drop-out and to missing observations at a particular time point.

Aim 2: Mediation models will be tested using regression analyses that estimate the path coefficients in the model and generate bootstrap confidence intervals (percentile, bias-corrected, and bias-corrected and accelerated) for total and specific indirect effects of the predictor (parenting behaviour) *on* the outcome variable (child brain development) through the mediator variable (indices of HPA function) [83]. This approach adjusts all paths for the potential influence of covariates not proposed to be mediators in the model.

Aim 3*:* The moderating and/or mediating effect of genetic and immune markers on associations between parenting behaviours and late childhood brain development will be assessed using regression models and boostrapping procedures as described for Aim 2.

Aim 4: Regression and path analyses will be used to assess if and how the environmental and biological factors measured are associated with child mental health.

## Discussion

This study will address four key gaps in current knowledge.

The first relates to the *lack of knowledge about sensitive periods of brain development* beyond early life. To this end, the late childhood period is especially important to consider, given that, as noted, this period is characterised by a wave of marked brain reorganization that continues over adolescence, and is second only to infancy in its extent. Up until very recently, it was thought that a wave of mass brain growth and reorganisation occurred around puberty, whereby brain systems matured rapidly in order to achieve adult configuration. However, more recent research shows that this ‘wave’ of brain development happens earlier, in mid- to late-childhood. For example, while early studies suggested a peak in the inverted-U shaped trajectory of frontal grey matter volumetric development during puberty (i.e., age 11 for girls and 12 for boys) [84], more recent and methodologically sophisticated studies suggest that the peak may occur earlier in development (i.e., before age 10) [85].

The second gap in knowledge relates to *the effects of adverse caregiving environments (including parenting) on brain development over time.* As mentioned above, studies investigating maltreatment in adult populations have found that early childhood maltreatment is associated with quite different effects on brain structure and function than are seen in youth maltreated in early childhood [34, 35]. This highlights that the effects of family environments on the brain may not be static but likely change across the life span. Indeed, we have shown that parenting is associated with longitudinal brain change during adolescence [1]. Further longitudinal research is crucial for understanding how the neurobiological effects of adverse family environments might change or unfold over time, from childhood to adulthood.

The third gap in knowledge is that *we know relatively little about how positive parenting affects child brain development.* We have provided evidence that positive parenting is associated with favourable child outcomes in terms of adjustment and mental health [25]. Some evidence from animal research shows that positive early life environments affect the brain in a pattern opposite to that typical of adverse environments. For example, animals raised in complex, enriched environments have more synapses in certain parts of their brains compared to animals raised in non-enriched environments [86]. Our recent human work has shown that aspects of positive parenting predict changes in brain structure over time during adolescence [4]. Further similar work is needed in different age periods, including childhood.

Finally, *we do not know the mechanisms linking caregiving environments with altered child brain development.* Alterations in stress reactivity in the HPA axis are a particularly plausible candidate [87], with substantial evidence indicating that children who are exposed to early adverse experiences, such as abuse [88], orphanage rearing [89], or low maternal care [90, 91] have increased cortisol reactivity. Basal cortisol levels have also been implicated, but findings have been inconsistent in regard to the direction of association. Further, levels of DHEA and its sulfate, DHEA-S (which are also released by the HPA axis and have anti-glucocorticoid [92] and neuroprotective [93] properties), have been consistently associated with childhood maltreatment and poor health outcomes [94]. The hippocampus, amygdala, pituitary gland and PFC represent key regions that are closely linked with the activity of the HPA axis. For example, while the hippocampus and PFC are known to mediate an inhibitory effect of glucocorticoids on stress-induced HPA activity [37], the amygdala is thought to be critical in activating the HPA axis in response to threat [36]. Despite these known links, there is currently limited work that has investigated associations between HPA axis function and brain structure in young individuals [95–97].

This study, by examining the neurobiological and behavioural consequences of variations in parenting in late childhood, has the potential to profoundly advance our understanding of child development and risk processes. Work on preventive interventions suggests the feasibility of intervening in the family context [98], but the further development of such interventions is now limited by our understanding of how parenting interacts with the brain development and the broader environment of young people to generate health problems.

## Additional files

Additional file 1: STROBE Cohort Study Checklist. (DOC 89 kb)

Additional file 2: Families and Childhood Transitions Study (FACTS) Detailed Measures File. (PDF 875 kb)

## Abbreviations

BMI: Body Mass Index
CRP: C-reactive Protein
DHEA: Dehydroepiandrosterone
DHEA-S: Dehydroepiandrosterone Sulphate
FACTS: Families and Childhood Transitions Study
FIT: Family Interaction Task
HPA: Hypothalamic-Pituitary-Adrenal axis
HPG: Hypothalamic-Pituitary-Gonadal axis
MRI: Magnetic Resonance Imaging
PFC: Prefrontal Cortex
PICF: Participant Information and Consent Form
RCH: The Royal Children’s Hospital
SIgA: Secretory Immunoglobulin-A
