# Supplementary material for: Study protocol: families and childhood transitions study (FACTS) – a longitudinal investigation of the role of the family environment in brain development and risk for mental health disorders in community based children
Source: BMC Pediatr. 2017 Jun 30;17:153. doi: 10.1186/s12887-017-0905-x (PMC5493854; doi:10.1186/s12887-017-0905-x)
Supplement: Supplementary file 2 — Families and Childhood Transitions Study (FACTS) Detailed Measures File. (PDF 875 kb) [file 12887_2017_905_MOESM2_ESM.pdf]

Families and Childhood Transitions Study Protocol Paper

Supplementary Materials - Detailed Measures

Table of Contents

Measures associated with the Mock MRI procedure 2
Image Acquisition 3
Structural Scan 3
Diffusion Weighted Images (DWI) 3
Resting fMRI 3
Affective faces fMRI task 3
Family Interaction Task: Event Planning Interaction & Problem Solving Interaction 4
Coding of Family Interaction Task Audio-visual data: Family Interaction Macro-coding System (FIMS) 5
Saliva Samples 7
Hair Sample 9
Anthropometry 10
Intelligence Quotient Test 11
Wechsler Intelligence Scale for Children – Version IV (WISC-IV) selected subtests 11
Silent Films Task (Phase 2 only) 12
Parent Interviews 13
Demographics and Health Information 13
Parent Questionnaires 14
Self-Report Questionnaires 14
Alabama Parenting Questionnaire (APQ) 14
The Adult Rejection Sensitivity Questionnaire (ARSQ) 15
Alcohol, Smoking and Substance Involvement Screening Test (ASSIST) 17
Beck Anxiety Inventory (BAI) 19
Centre for Epidemiologic Studies Depression Scale (CES-D) 19
Composite Abuse Scale (CAS) 21
Conflict Behaviour Questionnaire (CBQ) 22
Coping with Children's Negative Emotions Scale (CCNES) 22
Parental Reactions to Children's Positive Emotions Scale (PRCPS) 23
Revised Dyadic Adjustment Scale (RDAS) 25
Parent-Report on Child Questionnaires 26
Children's Depression Inventory 2 – Parent Report (CDI-2-PR) 26
Child Health Questionnaire (CHQ) 27
Children's Report of Parental Behaviour Inventory—Parent Report (CRPBI-PR) 29
The Pubertal Development Scale - Parent Report (PDS-PR) 30
The Sexual Maturity Status – Parent Report (SMS-PR) 31
The Child Behaviour Checklist (CBCL) 31
The Lifetime Incidence of Traumatic Events Parent Report (LITE-PR) 32
The Multidimensional Neglectful Behaviour Scale – Parent Report (MNBS-PR) 34
Child Questionnaires 35
Attachment Questionnaire for Children (AQC) 35
Brief Multidimensional Students Life Satisfaction Scale (BMSLSS) [Phase 2 only] 36
The Children's Depression Inventory 2 (CDI-2) 37
The Children's Rejection Sensitivity Questionnaire (CRSQ) 37
Four-Component Empathy Questionnaire for Children (Phase 2 only) 38
Edinburgh Handedness Inventory (EHI) 39
Kerns Security Scale (KSS) 40
The Spence Children's Anxiety Scale (SCAS) 40
Teacher Questionnaires 41
Social Skills Improvement System (SSIS) – Teacher Report 41

Detailed Measures

Measures associated with the Mock MRI procedure

Prior to completing the MRI, participants took part in a mock MRI procedure on a disused machine at the Royal Children's Hospital (RCH) in Melbourne. Mock MRI procedures improve both child MRI participation and the quality of structural/functional brain data collected during scans (de Bie et al., 2010; Carter, Greer, Gray & Ware, 2010). During the mock MRI, participants were shown photographs depicting each step of the MRI procedure which the researcher discussed with the child. After this, participants completed a visual analogue scale (VAS) to gauge whether they felt nervous, upset, interested and/or happy. For each emotion, participants were presented with a horizontal line bookended by the words 'Not at all' and 'A lot' and thus representing a continuum. Participants were then asked to draw an X at the point on the line which best represented their feelings toward completing the MRI. The purpose of this scale was to assist the researcher in understanding the child's feeling towards the MRI procedure allowing any issues to be worked through during the mock MRI. The researcher then showed the participant the mock MRI machine and encouraged them to practice lying in the machine while listening to a recording of sounds that an MRI machine typically makes while functioning. Furthermore, the participant tried wearing headphones and a helmet similar to those used in the actual MRI.

Carter, A.J., Greer, M.L.C., Gray, S.E., & Ware, R.S. (2010). Mock MRI: reducing the need for anaesthesia in children. *Pediatr Radiol, 40*:1368-74.

de Bie, H.M., Boersma, M., Wattjes, M.P., Adriaanse, S., Vermeulen, R.J., Oostrom, K.J., Huisman, J., Veltman, D.J., Delemarre-Van de Waal, H.A. (2010). Preparing children with a mock scanner training protocol results in high quality structural and functional MRI scans. *Eur J Pediatr, 169*(9):1079-85.

Image Acquisition

Neuroimaging data were acquired on the 3T Siemens TIM Trio scanner (Siemens, Erlangen, Germany) at the Murdoch Childrens Research Institute (MCRI), RCH, Melbourne, Australia. Participants lay supine with their head supported in a 32-channel head coil.

<u>Structural Scan</u>

T1-weighted images are acquired with motion correction (MPRAGE MoCo, repetition time = 2530msec; echo time1 = 1.74msec, echo time2 = 3.6msec, echo time3 = 5.46msec, echo time4 = 7.32msec; flip angle = 7°, field of view = 256x256 mm^2), which produced 176 contiguous 1.0mm thick slices (voxel dimensions=1.0mm^3). Sequence duration 5:19min.

<u>Diffusion Weighted Images (DWI)</u>

Diffusion weighted images are acquired (50 directions, b=3000 s/mm^2, 5 x b0 reference image, repetition time = 8500msec; echo time = 112msec; slices = 58; voxels = 2.3mm^3). In addition, reversed phase encoding scans ("Blip Up/Blip Down") with same voxel parameters are acquired to assist with correction of spatial and intensity distortion. Total sequence duration 8:00min.

<u>Resting fMRI</u>

A continuous functional gradient-recalled acquisition sequence is conducted at rest to acquire 154 whole-brain T2*-weighted echo-planar volumes (repetition time = 2400ms, echo time = 35ms, flip angle = 90°; field of view = 210 x 210mm^2, 38 interleaved slices, voxel size of 3.3mm^3). Complex field maps are obtained in order to correct for distortion caused by magnetic field inhomogeneities. Total sequence duration 6.18 min.

<u>Affective faces fMRI task</u>

Participants are administered (at the 18-month follow-up only) a modified version of the emotional face-matching task originally reported by Hariri et al. (2000). In this task participants must either match the gender of faces presented (face condition), or match shapes

(control condition). During each 4s “face trial”, participants are presented with a target face (centre top) and two probe faces (bottom left and right) and are instructed to match the probe of the same gender to the target by pressing a button either on the left or right. During each 4s “shape trial” participants are presented with a target shape (centre top) and two probe shapes (bottom left and right) and are instructed to match the probe of the same shape to the target by pressing a button either on the left or right. Each block consists of six consecutive trials containing angry or fearful faces (face condition) or shapes (control condition). A total of three 24-s blocks of each emotional face condition (i.e. angry and fearful) and six 24-s blocks of the control condition (shapes) are presented interleaved in a pseudo-randomized order. A fixation cross lasting 10-s is interspersed between each block. The total task time is 7 minutes. For each trial, response accuracy and response latency (reaction time) is obtained. Prior to the scan, participants complete a short practice version of the task with different emotional faces (happy and angry). Parameters include 136 whole-brain T2*-weighted echo-planar images (repetition time = 3000ms, echo time = 35ms, flip angle = 85°) within a field of view of 216x216mm^2, with a voxel size of 3mm^3. Forty interleaved slices are acquired. Total sequence duration 6:42min

Hariri, A. R., Bookheimer, S. Y., & Mazziotta, J. C. (2000). Modulating emotional responses: effects of a neocortical network on the limbic system. *Neuroreport, 11*(1), 43-48.

Family Interaction Task: Event Planning Interaction & Problem Solving Interaction

The Family Interaction Task (FIT) included two 15-minute tasks, an Event Planning Interaction (EPI) followed by a Problem Solving Interaction (PSI), that mother-child dyads completed together. During the EPI, participants planed three fun events together such as ‘taking a trip or vacation’. These activities were chosen from the Pleasant Events Checklist (PEC), a modified version of the Pleasant Event Schedule (MacPhillamy & Lewinsohn,

1976). During the PSI, participants chose three conflict-eliciting issues from the Issues Checklist (IC) such as ‘talking back to parents’ (Prinz, Foster, Kent & O’Leary, 1979). The dyads then problem solved each issue in detail. These conversations were video recorded using a separate camera and microphone for each participant.

Macphillamy, D. J., & Lewinsohn, P. M. (1982). The Pleasant Events Schedule: Studies on Reliability, Validity, and Scale Intel-correlation. *Journal of Consulting and Clinical Psychology*, *50*(3), 363–380.

Prinz, R. J., Foster, S., Kent, R. N., & O’Leary, K. D. (1979). Multivariate Assessment of Conflict in Distressed and Nondistressed Mother-Adolescent Dyads. *Journal of Applied Behavior Analysis*, *12*(4), 691–700. doi:10.1901/jaba.1979.12-691P

Coding of Family Interaction Task Audio-visual data: Family Interaction Macro-coding System (FIMS)

Audio-visual material recorded during the family interaction tasks was coded using the Family Interaction Macro-coding System (FIMS; Holmbeck, Zebracki, Johnson, Belvedere & Hommeyer, 2007). FIMS is a global coding method (Holmbeck et al 1995) adapted from a system devised by Smetana et al (1991). Coders viewed each video and then provided 5-point likert scale ratings on 67 items representing various dimensions designed to assess parent, child and family behaviour. FIMS items are outlined in a coding manual grouped under sections targeting interaction style, conflict, affect, control, parental behaviours and collaborative problem solving, and general family measures (Holmbeck et al., 2003).

Two researchers within our lab were trained by experienced coders from the laboratory of Professor Grayson N. Holmbeck at Loyola University of Chicago. Training included a tutorial demonstrating how to apply each FIMS item to video material, scoring of

several training videos and a final reliability assessment for which trainees independently scored five videos using FIMS codes. Trainees were required to achieve 90% agreement with from a master document for each training video. Once this level of proficiency was achieved coding commenced. The coders worked systematically through the audiovisual material in a counterbalanced sequence; participant dyads were coded in batches of 20 with one coder starting at each end of the set. For the first 60 participants reliability was calculated between coders three times. At the completion of all coding reliability was calculated again with our coders achieving 95% agreement between codes.

FIMS items: excluded & additional items – The FIMS system includes general family measures designed for interactions involving more than two people (i.e. two parents and a child). Interactions for our study only involved mother-child dyads so most of these items were excluded excepting the item ‘the family is able to reach agreement or resolution’ which was included because none of the dyadic items covered this behaviour. Also, the FIMS has been used to assess parenting of children with chronic medical conditions with items such as ‘active catering to the child’ and ‘parental behaviour that infantalises the child’ being particularly relevant to such as focus. We excluded these items because the participants in our study represented a healthy population for whom these items were not relevant.

For the purposes of this study minor additional items were also added to the original FIMS measure. Under the Affect section of FIMS, item U codes for ‘intensity of positive affect expression/emotionality’ – this item codes for both intensity and frequency of positive affect – and item V codes for frequency of positive affect. Our coders retained these items and for balance added a new item to code purely for intensity of positive affect excluding frequency of positive affect. In relation to negative affect, original FIMS codes included item X ‘Frequency of negative affect’. We retained this item but added an additional four items to

code for 1) intensity and 2) frequency of aggressive affect, and 3) intensity and 4) frequency of dysphoric affect respectively.

Holmbeck, G. N., Belvedere, M., Gorey-Fergson, L., & Schneider, J. (1995). *Manual for family macro-coding*. Chicago: Loyola University.

Holmbeck, G. N., Westhoven, V. C., Phillips, W. S., Bowers, R., Gruse, C., Nikolopoulos, T., . . . Davison, K. (2003). A multimethod, multi-informant, and multidimensional perspective on psychosocial adjustment in preadolescents with spina bifida. *J Consult Clin Psychol, 71*(4), 782-796.

Holmbeck, G. N., Zebracki, K., Johnson, S. Z., Belvedere, M., & Hommeyer, J. S. (2007). *Parent-child interaction macro-coding manual*. Chicago: Loyola University.

Smetana, J. G., Yau, J., Restrepo, A., & Braeges, J. L. (1991). Adolescent-parent conflict in married and divorced families. *Developmental Psychology, 27*, 1000–1010.

Saliva Samples

Short-term circulating levels of cortisol, testosterone, DHEA and DHEA-S were obtained through the measurement of average salivary levels across two mornings. DHEA and DHEA-S are hormonal markers of adrenarcheal development, testosterone of adrenarcheal development in girls and both adrenarcheal and gonadarcheal development in boys, and cortisol an important corollary of HPA axis functioning. With the help of a parent or guardian, children collected two saliva samples: one was collected the day before their appointment and the second was collected on the day of their appointment. All equipment was provided. Participants were instructed to collect the sample immediately after waking (prior to tooth brushing, eating, drinking or taking medication). Saliva was collected via the passive drool of whole saliva through a straw into the test tube. Parents were asked to time the

duration of the 2ml saliva collection using a stopwatch. Samples were then frozen with Techni-Ice™ in the participant's freezer within a sealed container. This was then transported to RCH on the day of the appointment in a cooler bag. During the assessment the sample was stored in a larger Styrofoam cooler box packed with Techni-Ice™, this was later transferred to the MCRI freezer and stored at -30°C. Families were asked to minimize the time that the samples spent out of the freezer. At time of assay (at the conclusion of T1 data collection), samples were defrosted and centrifuged, with the supernatant assayed for levels of testosterone, cortisol, DHEA and DHEA-S. T1 assays were conducted at MCRI, using Salimetrics ELISA kits of the same lot number, and with in-house controls. The inter-assay coefficients of variation (CVs) for the baseline assessment were: DHEA = 11.76%; DHEA-S = 13.77%; testosterone = 10.47%; cortisol = 5.32%. The intra-assay CVs were: DHEA = 9.03%; DHEA-S = 7.82%; testosterone = 8.17%; cortisol = 3.47%.

Remaining supernatant is stored in 1ml aliquots (typically x3) in a -80°C freezer for future assays when funding allows, including other hormones (e.g., oestradiol) and immune system biomarkers (e.g., C-reactive protein and secretory immunoglobulin A). Salivary assays of biomarkers testosterone, cortisol, DHEA, and DHEA-S, are well-accepted substitutes for measuring serum levels (Byrne et al., 2013; Granger et al., 2007), although there are methodological idiosyncrasies for each (e.g., DHEA-S, see Simmons et al., 2014).

Byrne, M. L., O'Brien-Simpson, N. M., Reynolds, E. C., Walsh, K. A., Laughton, K., Waloszek, J. M., . . . Allen, N. B. (2013). Acute phase protein and cytokine levels in serum and saliva: a comparison of detectable levels and correlations in a depressed and healthy adolescent sample. *Brain Behav Immun, 34*, 164-175.

Granger, D. A., Kivlighan, K. T., Fortunato, C., Harmon, A. G., Hibel, L. C., Schwartz, E. B., & Whembolua, G. L. (2007). Integration of salivary biomarkers into developmental

and behaviorally-oriented research: problems and solutions for collecting specimens. *Physiol Behav, 92*(4), 583-590.

Simmons, J. G., Whittle, S. L., Patton, G. C., Dudgeon, P., Olsson, C., Byrne, M. L., . . . Allen, N. B. (2014). Study protocol: imaging brain development in the Childhood to Adolescence Transition Study (iCATS). *BMC Pediatr, 14*, 115-124.

Hair Sample

Hair samples were collected to index longer-term hormone levels (Russell, Koren, Rieder, & Van Uum, 2012; Simmons et al., 2015), primarily cortisol, DHEA and testosterone. A section of hair approximately 1cm^2 surface area on the scalp was taken from the posterior vertex. Longer samples were tied with string and the scalp-end of the sample was clearly marked, while shorter samples were stored untied in an envelope. Samples were kept in controlled conditions away from light and extreme temperatures. Hair grows at a rate of approximately 1cm per month (Wenning, 2000), therefore a section of hair that is 3cm in length provides an indication of hormonal output over several months. The sample was taken from the posterior vertex of the scalp as it has the lowest coefficient of variation for hormonal levels compared with other areas of the scalp (Sauvé, Koren, Walsh, Tokmakejian, & Van Uum, 2007). A maximum length of 3cm of hair was analysed to reduce damage to the hair from washing and sun exposure (Meyer & Novak, 2012). Hair assays for T1 were conducted by Stratech Scientific and processed and assayed as described previously (Davenport, Tiefenbacher, Lutz, Novak, & Meyer, 2006 #2940), using Salimetrics ELISA kits for cortisol, DHEA and testosterone. The intra-assay coefficient of variation (CV) for T1 was 5.1%, and inter-assay CV 5.8%.

Meyer, J. S., & Novak, M. A. (2012). Minireview: Hair cortisol: a novel biomarker of hypothalamic-pituitary-adrenocortical activity. *Endocrinology, 153*(9), 4120-4127.

Russell, E., Koren, G., Rieder, M., & Van Uum, S. (2012). Hair cortisol as a biological marker of chronic stress: current status, future directions and unanswered questions. *Psychoneuroendocrinology, 37*(5), 589-601.

Simmons, J. G., Badcock, P. B., Whittle, S. L., Byrne, M. L., Mundy, L., Patton, G. C., . . . Allen, N. B. (2015). The lifetime experience of traumatic events is associated with hair cortisol concentrations in community-based children. *Psychoneuroendocrinology, 63,* 276-281.

Sauvé, B., Koren, G., Walsh, G., Tokmakejian, S., & Van Uum, S. H. M. (2007). Measurement of cortisol in human hair as a biomarker of systemic exposure. *Clinical and Investigative Medicine, 30*(5), 183-192.

Wenning, R. (2000). Potential problems with the interpretation of hair analysis results. *Forensic Science International, 107,* 5-11.

Anthropometry

Body composition of the child was obtained through measurements of height, weight and waist circumference as described in Simmons (Simmons et al., 2014). Two measurements of each were taken, however if they were not within a specified range (0.5cm for height, 0.1 kg for weight, 0.5cm for waist) a third measurement was collected. The mean value was calculated for two measurements; the median value if three measurements were obtained. Raw figures (either the mean or median) were transformed into z scores according to age and gender related charts—these values were used to indicate body composition, a validated indicator for obesity across childhood and adolescence (Cole, Bellizzi, Flegal, & Dietz, 2000). Waist circumference was used to indicate intra-abdominal fat—this has been validated as a

reliable indicator (Taylor, Jones, Williams, & Goulding, 2000). Measurements were taken using the following materials: a portable rigid Invicta stadiometer was used to measure height; calibrated Tanita THD 382 digital scales measured weight; and waist circumference was measured by non-stretch anthropometric tape (according to the International Society for the Advancement of Kinanthropometry (ISAK) protocols). To improve accuracy of these measurements children were instructed to remove bulky or heavy items such as shoes and coats.

Cole, T. J., Bellizzi, M. C., Flegal, K. M., & Dietz, W. H. (2000). Establishing a standard definition for child overweight and obesity worldwide: international survey. *BMJ, 320*(7244), 1240-1243.

Simmons, J. G., Whittle, S. L., Patton, G. C., Dudgeon, P., Olsson, C., Byrne, M. L., et al. (2014). Study protocol: Imaging brain development inthe Childhood to Adolescence Transition Study(iCATS), *14*(1), 1–10.

Taylor, R. W., Jones, I. E., Williams, S. M., & Goulding, A. (2000). Evaluation of waist circumference, waist-to-hip ratio, and the conicity index as screening tools for high trunk fat mass, as measured by dual-energy X-ray absorptiometry, in children aged 3-19 y. *Am J Clin Nutr, 72*(2), 490-495.

Intelligence Quotient Test

<u>Wechsler Intelligence Scale for Children – Version IV (WISC-IV) selected subtests</u>

Several subtests from the Wechsler Intelligence Scale for Children – Version IV (WISC-IV; Australian Language Adaptation edition) (Wechsler, 2003) were used, specifically matrix reasoning, vocabulary and symbol search, in order to give a brief estimate of intelligence.

Norms are based on 851 children and adolescents, aged 6 years to 16 years and 11 months, who participated in the Australian Standardisation Project.

Wechsler, D. (2003). Wechsler intelligence scale for children–Fourth Edition (WISC-IV). *San Antonio, TX: The Psychological Corporation*.

Silent Films Task (Phase 2 only)

Silent films is a video-based task developed by (Devine & Hughes, 2013). It assesses cognitive empathy (theory of mind). This video based task is designed to be an analogue of Happé's Strange Stories task (Happé, 1994; White et al., 2009). It is comprised of five short film clips (mean length of 25s) from a classic silent film: Harold Lloyd's comedy Safety Last (Roach, Newmeyer, & Taylor, 1923). The clips depict instances of deception, false belief, belief-desire reasoning, and misunderstanding. The task requires participants to use their understanding of beliefs and desires to explain the behaviour of characters in scenarios. For example, in one clip, the main character becomes accidentally locked in the back of a van by the driver. The child is asked, "Why do you think the driver locks Harold in the van?" Responses are given 0, 1 or 2 points depending on the understanding the child shows – 2 points for full understanding, 1 point for partially correct, and 0 for incorrect. The use of silent film clips broadens the task's applicability for use with different language groups and with children of low verbal ability. It has been validated in 8-13 year olds and has good psychometric properties (Devine & Hughes, 2015).

Devine, R. T., & Hughes, C. (2013). Silent films and strange stories: theory of mind, gender, and social experiences in middle childhood. *Child Development, 84*(3), 989-1003.

Devine, R. T., & Hughes, C. (2015). Measuring theory of mind across middle childhood: Reliability and validity of the Silent Films and Strange Stories tasks. *Journal of Experimental Child Psychology*.

Happé, F. G. E. (1994). An advanced test of theory of mind: Understanding of story characters' thoughts and feelings by able, mentally handicapped and normal children. *Journal of Autism and Developmental Disorders*, 24, 129–154.

Roach, H. (Producer), Newmeyer, F. C. (Director), & Taylor, S. (Director). (1923*). Safety last!* [motion picture]. Culver City, CA: Hal Roach Studios.

White, S., Hill, E., Happé, F., & Frith, U. (2009). Revisiting the Strange Stories: Revealing mentalising impairments in autism. *Child Development*, 80, 1097–1117.

Parent Interviews

Demographics and Health Information

Detailed demographic information was collected including parental age, language spoken at home, race, ethnicity, adoption status, and country of birth for the maternal and paternal grandparents, mother, father, and child. Also collected was socioeconomic data such as residential neighbourhood, parental education, occupation and annual household income. Information about family structure was collected including significant caregivers and siblings (both biological and non-biological) living inside as well as outside the home. A brief mental health history of the primary caregivers was taken using the maternal-reported lifetime diagnosis of psychiatric symptoms – a brief interview using the dedicated subsection of the Kiddie-Schedule for Affective Disorders and Schizophrenia-Present and Lifetime Version (K-SADS-PL; Kaufman et al., 1997). During this interview, the parent was asked to recall whether they had been diagnosed, or experienced symptoms relating to, the following presentations: depression, anxiety, mania/hypomania, schizophrenia, psychotic symptoms, conduct or antisocial disorders, and substance use. If mental health diagnosis/symptoms were

endorsed, parents were asked whether treatment was received and if so what type – counselling, medication, etc. Information pertaining to the physical health of the child and primary maternal figure is also gathered for the purpose of MRI safety exclusions. A more extensive medical history was taken for the child, for the purpose of eligibility and exclusions, which included: chronic and recent illnesses, current and previous medications, developmental disorders and stressful events experienced 3 months prior to the assessment.

Kaufman, J., Birmaher, B., Brent, D., Rao, U., Flynn, C., Moreci, P., . . . Ryan, N. (1997). Schedule for Affective Disorders and Schizophrenia for School-Age Children-Present and Lifetime Version (K-SADS-PL): Initial Reliability and Validity Data. *Journal of the American Academy of Child & Adolescent Psychiatry, 36*(7), 980-988.

Parent Questionnaires

Questionnaires completed by parents/guardians comprised self-report and reports about the child. The broad domains covered by questionnaires were; parenting and family environment, symptoms and behaviours relating to the parent, and symptoms, traumatic events, adjustment and development relating to the child.

Self-Report Questionnaires

<u>Alabama Parenting Questionnaire (APQ)</u>

The APQ is a 42-item scale, comprising five dimensions of parenting relevant to the aetiology and treatment of child externalising and internalising problems, including: (1) positive involvement, (2) supervision and monitoring, (3) positive discipline, (4) consistency in discipline and (5) corporal punishment (Shelton, Frick & Wootton, 1996). The parent answers based on the previous six months and responses are rated on a 5-point Likert scale based on

the frequency of behaviour, 1= never to 5 = always (Shelton, Frick & Wootton, 1996). Factor analyses have substantiated the five factor subscales (Essau, Sasagawa, & Frick, 2006), and the questionnaire has been utilised and published widely with a range of populations. The APQ has normative data in the relevant age range, as well good evidence for reliability and validity (e.g., Dadds, Maujean, & Fraser, 2003).

Shelton, K. K., Frick, P. J., & Wootton, J. (1996). Assessment of parenting practices in families of elementary school-age children. *Journal of clinical child psychology, 25*(3), 317-329.

Dadds, M. R., Maujean, A., & Fraser, J. A. (2003). Parenting and conduct problems in children: Australian data and psychometric properties of the Alabama Parenting Questionnaire. *Australian Psychologist, 38*(3), 238-241.

<u>The Adult Rejection Sensitivity Questionnaire (ARSQ)</u>

R*ejection sensitivity* refers to the tendency to anxiously expect rejection, readily perceive and overreact to it. The questionnaire presents nine different scenarios, and in particular interpersonal events with a significant other, friend or family member, in which the respondents have to imagine themselves. For each situation two questions are presented. The first one investigates the level of concern about the outcome of that situation; response options are on a Likert-scale from 1 (very unconcerned) to 6 (very concerned). The second question asks how likely they think the situation would have an outcome of acceptance rather than rejection. Response options are again on a Likert-scale from 1 (very unlikely) to 6 (very likely).

Analyses on the original questionnaire revealed a high reliability either internal (Cronbach's alpha = .83) and test-rest (.83 after 3 weeks and .78 after 4 months - tested on two different subsamples) and in a series of experiments it demonstrated a sound predictive

validity. The questionnaire also showed a good convergent and divergent validity with measures of construct theoretically related (e.g. it was negatively correlated with self-esteem and secure attachment). It has been reported elsewhere that the adult modified version correlates with the original (r = .87) and has sufficient reliability (Cronbach's alpha = .74) however the original data are unpublished (Downey et al., 2006). Further investigation of the psychometric properties of the ARSQ (Innamorati et al, 2014) challenged the one-factor model originally proposed to support the presence of a general factor (rejection sensitivity) and two group factors (rejection anxiety and rejection expectancy). In this study the both the general factor (Cronbach's alpha = .82) and the two group factors (Cronbach's alpha = 0.82 and 0.78) showed good internal consistency Both the general factor and the group factors correlated significantly and positively with measures of depression, anxiety and hopelessness measured respectively with the Beck Depression Inventory-II, the Beck Anxiety Inventory and the Beck Hopelessness Scale.

Downey G, Feldman SI (1996) Implications of rejection sensitivity for intimate relationships. Journal of Personality and Social Psychology. 70(6) 1327-1343

Downey G, Berenson KR, Kang J (2006). Correlates of the Adult Rejection Sensitivity Questionnaire, Columbia University. Unpublished data.

Innamorati M, Balsamo M, Fairfield B, Fabbricatore M, Tamburello A, Saggino A (2014) Construct Validity and Reliability of the Adult Rejection Sensitivity Questionnaire: A Comparison of Three Factor Models. Depression Research and Treatment, vol. 2014, Article ID 972424, 10 pages.

Alcohol, Smoking and Substance Involvement Screening Test (ASSIST)

The ASSIST (version 3.1) is a questionnaire originally developed by the World Health Organization (WHO) as a tool for early screening of psychoactive substance use in primary health care setting. The questionnaire investigates the use of substances, not for medical purpose, across the life span and in the three months preceding the assessment. The substances listed are alcohol, tobacco, cannabis, cocaine, amphetamine type stimulants, inhalants, sedative or sleeping pills, hallucinogens, opioids and other - as specified by the respondent. There are 8 questions in total, which assess use, frequency, desire and urge to use (retrospective report), and functional impacts. Response options for Question 2 to 5 are never (not used in the last 3 month), once or twice (1 – 2 times in the last 3 month), monthly (1 – 3 times in each month, of the last 3 month), weekly (1 – 4 times per week, over the last 3 months), daily or almost daily (5 – 7 days per week, over the last 3 months). Questions 6 and 7 refer back to substances endorsed in Question 1 (lifetime use) asking how often a friend, relative or anyone else has ever expressed concern about the use of the substance, and any failure tentative to control, cut down or stop using the substance. Response options for Questions 6 and 7 are No – never, yes – in the past three months, yes – but not in the past three months. Question 8 asks about any use of drug by injection, not for medical purpose. Response options for question 8 are No – never, yes – in the past three months, yes – but not in the past three months.

The first version of the ASSIST was released in 1997 and it initially consisted in a 12 items questionnaire. In Phase I of the project reliability analyses were conducted and the ASSIST showed good test-retest reliability (WHO ASSIST Working Grp, 2002). The test-retest kappas averaged across questions ranged from .90 (Q1 – “ever used”) to .58 (Q7 – “feelings of regret” – question subsequently dropped). The average kappas by substance classes ranged from .61 (sedatives) to .78 (opioids). After the statistical analyses and the

feedback of participants the ASSIST was subsequently revised. 4 questions were dropped and others rephrased resulting in an 8 items questionnaire (version 2.0) to ensure clarity, feasibility and relevance of the questions. In Phase II the ASSIST 2.0 was administered in different conditions and settings in 7 different countries and further analyses were carried (Humeniuk et al., 2008). The questionnaire so formulated showed a good concurrent validity, demonstrated by significant correlations with other instruments of screening such as the Addiction Severity Index (ASI), the Severity of Dependence Scale (SDS) and the MINI International Neuropsychiatric Interview (MINI-Plus). It demonstrated a good internal consistency (Cronbach's alpha) that was .89 for the total score (i.e. the sum of response across all substances) and ranged from .77 to .94 considering each substance separately. The questionnaire demonstrated also to be able to discriminate between "use" (low risk), "abuse" (moderate risk) and "dependence" (high risk) groups. Therefore cut-off scores were created resulting is the current version (3.1 for clinical purpose; Humeniuk et al., 2010).

WHO ASSIST Working Group (2002) The Alcohol, Smoking and Substance Involvement Screening Test (ASSIST): Development, Reliability and Feasibility. *Addiction*. 97:1183-1194.

Humeniuk RE, Ali RL, Babor TF, Farrell M, Formigoni ML, Jittiwutikarn J, de Lacerda RB, Ling W, Marsden J, Monteiro M, Nhiwatiwa S, Pal H, Poznyak V, Simon S (2008) Validation of the alcohol, smoking and substance involvement screening test (ASSIST). *Addiction*. 03(6):1039-1047.

Humeniuk RE, Henry-Edwards S, Ali RL, Poznyak V, Monteiro M (2010). *The Alcohol, Smoking and Substance Involvement Screening Test (ASSIST): manual for use in primary care*. Geneva, World Health Organization

Beck Anxiety Inventory (BAI)

The BAI scale is a self-report measure of severity of anxiety. It was originally develop with psychiatric population to overcome the lack of measures able to discriminate between anxiety and depression. It consists of 21 items that represent common symptoms of anxiety and tap on two main components (factors): somatic symptoms and subjective anxiety/panic symptoms. The respondent is required to rate how much they have been bothered by each symptom in the week preceding (including the day of) the assessment. The scale ranges from 0 to 3, where 0 corresponds to not at all, 1 to mildly ("it did not bother me much"), 2 to moderately ("it was very unpleasant but I could stand it") and 3 to severely ("I could barely stand it"). The scale takes about 5-10 minutes to complete.

The BAI was able to discriminate between patients with anxiety disorders (either primary or secondary) from depressed patients (without anxiety disorders) and controls. It showed high internal consistency (Cronbach's alpha = .92). Test-retest reliability assessed on subsample of patients after a week was .75. Convergent and discriminant validity were also tested and the BAI showed higher positive correlations with other measures of anxiety such as the Hamilton Anxiety Rating Scale for-Revised (r = .51) whereas very low correlations with measures of depression such as the Hamilton Rating Scale for Depression Revised (r = .21) or the Hopelessness Scale (r = .15). The correlation with the Beck Depression Inventory was still moderately high (r = .48).

Beck, A.T., Epstein, N., Brown, G., Steer, R.A. (1988) An inventory for measuring clinical anxiety: psychometric properties. Journal of Consulting and Clinical Psychology. 56:893–897

Centre for Epidemiologic Studies Depression Scale (CES-D)

The CES-D is a self-report measure originally developed to study the epidemiology of current

depressive symptomatology in the general population. The 20 items can be grouped to represent the major components of depression as defined by the literature, such as depressed mood, loss of interest (anhedonia), loss of appetite, sleep disturbance, feelings of guilt and worthlessness and psychomotor retardation. 4 of 20 items were phrased in a positive way to break the possible tendency to answer all the questions in a negative direction. Respondents are asked to rate "how often this past week did you…", response options range from 0 to 3 for each item (0 = rarely or not at all, 1 = sometimes, 2 = occasionally, 3 = most of the time). The total score ranges from 0-60 and a higher score indicates a greater symptomatology. It is widely adopted as clinical significance cut-off a total score equal to or above 16. Validation studies were conducted both on the general population and on two clinical subsamples. The internal consistency (Cronbach's alpha) ranged between .84 and .85 in general population and .90 in the clinical subsample. Test-retest reliability on general population ranged from .51 to .67 and from .32 to .54 depending on the method of re-test (self-administered mail-backs or re-interviews respectively). It is worth to consider that the scale is designed to measure the *current* level of symptomatology (the past week) therefore variability is expected. In the clinical sample correlation of the score at the admission and after 4 weeks of treatment was .53. The scale was also able to discriminate well between patients and general population and moderately between levels of severity in the patient group. Regarding convergent validity, in the clinical sample it showed moderate to high correlations with the Hamilton Clinician's Rating scale and the Rasking Rating scale (respectively .44 and .54 at admission and .69 and .75 after 4 weeks of treatment). It showed also positive correlation with other self-report scales on depression such as the Lubin and the Bradburn Negative Affect and negative correlations with self-report scales on positive affect like the Bradburn Positive Affect both in the general population and in the clinical sample, although they were higher in the latter. The authors run also a principal component factor analysis and found a 4 factors structure as

follow: depressed affect, positive affect, somatic and retarded activity and interpersonal. However, they recommended not adopting a factorial approach but considering the total score as index of symptomatology.

Radloff, L.S. (1977). The CES-D scale: A self-report depression scale for research in the general population. *Applied Psychological Measurement*. 1: 385-401

<u>Composite Abuse Scale (CAS)</u>

The CAS captures severity and frequency of a range of partner behaviors and comprises 30 items consisting of acts of physical, emotional, and sexual abuse (Hegarty & Valpied, 2013). Items are presented in a six point format requiring respondents to answer "never", "only once", "several times", "monthly", "weekly" or "daily" in a twelve month period. The four following factors have been validated in community and clinical studies: (1) severe combined abuse, emotional abuse, physical abuse, and harassment (Hegarty, Bush, & Sheehan, 2005; Hegarty & Valpied, 2013; Loxton et al., 2013). Internal consistency reliability has been reported as 0.85 or above and for the majority of sub-scales greater than 0.9, and the corrected item-total correlations as generally high (more than 0.5; Hegarty & Valpied, 2013).

Hegarty, K., Bush, R., & Sheehan, M. (2005). The Composite Abuse Scale: Further development and assessment of reliability and validity of a multidimensional partner abuse measure in clinical settings. *Violence and Victims, 20*(5), 529-547.

Hegarty, K., Valpied, J. (2013). *Composite Abuse Scale Manual*. Melbourne, Australia. University of Melbourne

Loxton, D., Powers, J., Fitzgerald, D., Forder, P., Anderson, A., Taft, A., Hegarty, K. The Community Composite Abuse Scale: Reliability and validity of a measure of intimate

partner violence in a community survey from the ALSWH (2013), *Journal of Women's Health, Issues & Care*, 2 (4).

Conflict Behaviour Questionnaire (CBQ)

The CBQ is a 20-item true/false self-report measure that assesses general conflict between parents and their children over a two week period. Items includes statements such as, ‘My child is easy to get along with’ and ‘My child often doesn’t do what I ask’. It has demonstrated sound discriminant validity (Grace, Kelley, & McCain, 1993) and adequate internal consistency (Robin & Foster, 1989). In addition, the CBQ has been found to discriminate between distressed and non-distressed families (Robin & Foster, 1989). The shortened 20-item version is highly correlated with the original 75-item questionnaire (.96; Prinz, Foster, Kent & O’Leary, 1979; Robin & Foster, 1989).

Grace, N. C., Kelley, M. L., & McCain, A. P. (1993). Attribution processes in mother-adolescent conflict. *Journal of Abnormal Child Psychology, 21*(2), 199-211.

Prinz, R. J., Foster, S., Kent, R. N., & O'Leary, K. D. (1979). Multivariate assessment of conflict in distressed and nondistressed mother-adolescent dyads. *Journal of Applied Behavior Analysis, 12*(4), 691-700.

Robin, A.L. & Foster, S.L. (1989). *Negotiating parent-adolescent conflict: A behavioral-family systems approach.* New York: Gilford Press.

Coping with Children’s Negative Emotions Scale (CCNES)

The CCNES presents 12 vignettes describing everyday situations involving children’s negative emotions (e.g., crying after losing a favorite possession; Fabes, Eisenberg, & Bernzweig, 1990). Parents rate their likelihood of responding to the child’s negative emotions

on a Likert-type scale from 1 (*very unlikely*) to 7 (*very likely*). The scale has demonstrated good construct validity, and includes six subscales which are often combined to form composite subscales of supportive reactions (expressive encouragement, emotion-oriented focus, and problem-oriented focus), and nonsupportive reactions (punishment, minimization, and personal distress; Fabes, Poulin, Eisenberg, & Madden-Derdich, 2002). Factor analysis of the CCNES has suggested there may be only four rather than six subscales (Fabes, Poulin, Eisenberg, & Madden-Derdich, 2002) and more recently an 'ignoring' subscale, with internal consistency, has been described (Mirabile, 2015).

Fabes, R.A., Eisenberg, N., Bernzweig, J.(1990) *Coping with Children's Negative Emotions Scale (CCNES): Description and Scoring*. Tempe, AZ: Arizona State University

Fabes, R. A., Poulin, R. E., Eisenberg, N., & Madden-Derdich, D. A. (2002). The Coping with Children's Negative Emotions Scale (CCNES): Psychometric properties and relations with children's emotional competence. *Marriage and Family Review, 34*(3-4), 285-310.

Mirabile, S. P. (2015). Ignoring Children's Emotions: A novel ignoring subscale for the Coping with Children's Negative Emotions Scale. *European Journal of Developmental Psychology, 12*(4), 459-471.

Parental Reactions to Children's Positive Emotions Scale (PRCPS)

The PRCPS aims to evaluate parental reaction to child's expression of positive emotions (Ladouceur, Reid & Jacques, 2002). It consists of 12 different scenarios in which the child is expressing a positive emotion in different ways, such as jumping, giggling or laughing. Along with that, different type of parental response are presented, which refer to 4 main styles: socialisation, encouragement, discomfort and reprimand. Parents are asked to rate their

likelihood of adopting each response on a Likert scale from 1 (very unlikely) to 7 (very likely). Mothers of children between age 4 and 7 constituted the original sample (Ladouceur, Reid & Jacques, 2002). A further study adapted the original version to an older sample of early adolescents 11-13 years old by modifying 5 of the 12 scenarios (Yap, Allen & Ladouceur, 2008). In this study correlational analyses were conducted and three subscales (socialisation, encouragement and discomfort) appeared to be positively correlated between each other and negatively correlated with the subscale encouragement. Therefore the three subscales were grouped together into one composite scale defined "invalidating reactions" opposite to encouragement that it supposes to reflect non-restrictive (validating) responses to positive affectivity. In the original sample the internal consistency of the subscales was good (Cronbach's alpha ranged between .72 an .82 at pre-test and .79 and .88 at post-test), while the test-retest reliability ranged from .60 to .79. The authors also provided information about convergent and divergent validity testing the correlations between the PRCPS subscales and other questionnaires focussed on the emotion communication in the family environment, such as the Coping with Children's Negative Emotions Scale (CCNES), the Self-Expressiveness in the Family Questionnaire (SEFQ) and the Family Environment Questionnaire (FEQ).

Ladouceur C, Reid L, & Jacques A (2002). Construction and validation of the Parents' Reaction to Children's Positive Emotions Scale. Canadian Journal of Behavioural Science. 31: 8 – 18.

Yap, M., Allen, N.B., & Ladouceur, C. (2008) Maternal Socialization of Positive Affect: The Impact of Invalidation on Adolescent Emotion Regulation and Depressive Symptomatology. Child Development. 79 (5) 1415 – 1431

Revised Dyadic Adjustment Scale (RDAS)

The RDAS is a 14-item scale designed to measure relationship satisfaction (Busby, Christensen, Crane, & Larson, 1995). Items one to six rate the approximate extent of agreement or disagreement between the participant and their partner on issues such as, religious matters and career decisions. Six responses are available, ranging from 'always agree' to 'always disagree'. The language of the response options changes for items seven to ten to 'all of the time' to 'never'. For item 11, 'Do you and your mates engage in outside interests together?', five responses are presented ranging from 'everyday' to never'. The final three items revert back to six responses, scored from 'Never' to 'More Often'. The RDAS is a revised version of the original 32-item Dyadic Adjustment Scale (Spanier, 1976). The revised version offers improved psychometric properties, is shorter, and includes only 3 of the original 4 subscales: (1) Dyadic Consensus – degree to which respondent agrees with partner; (2) Dyadic Satisfaction -- degree to which respondent feels satisfied with partner; (3) Dyadic Cohesion –degree to which respondent and partner participate in activities together (Busby, Christensen, Crane, & Larson, 1995). The psychometric properties of the RDAS have been validated internationally in samples of couples (e.g., Hollist et al., 2012, Turliuc & Muraru, 2013)

Busby, D. M., Christensen, C., Crane, D. R., & Larson, J. H. (1995). A revision of the dyadic adjustment scale for use with distressed and nondistressed couples: Construct hierarchy and multidimensional scales. *Journal of Marital and Family Therapy, 21*(3), 289.

Hollist, C. S., Falceto, O. G., Ferreira, L. M., Miller, R. B., Springer, P. R., Fernandes, C. L. C., & Nunes, N. A. (2012). Portuguese translation and validation of the revised dyadic adjustment scale. *Journal of Marital and Family Therapy, 38*, 348-58.

Spanier, G. B. (1976). Measuring Dyadic Adjustment: new scales for assessing the quality of marriage and similar dyads. *Journal of Marriage and Family, 38*(1), 15-28.

Turliuc, M. N., & Muraru, A. A. (2013). Psychometric properties of the revised Dyadic Adjustment Scale on a sample of married adults. *Journal of Psychological and Educational Research, 21*(1), 49-76.

Parent-Report on Child Questionnaires

Children's Depression Inventory 2 – Parent Report (CDI-2-PR)

The CDI-PR is a parent-report of the child's depressive symptoms. It has 17 items in total, with 4 reverse-scored items to avoid negative bias. Similar to the CDI-C, questions aim to identify the presence of internalising pathology across a number of areas including: emotionally, behaviorally, academically and socially. Each item is a statement about the parent's observations of their child across the past two weeks. Items are rated on a 4-point scale from "not at all (0)" "some of the time (1)", "often (2)", "much or most of the time (3)". Scored similar to the CDI, the higher the score, the greater presence of internalizing symptoms. The CDI-P has sound reliability and validity (Cole, Martin, Peeke, Seroczynski, & Hoffman, 1998; Romano & Nelson, 1988). When compared to the CDI-C, there is also evidence to show strong concurrent validity (Fristad, Weller, Weller, Teare, & Preskorn, 1991; Romano & Nelson, 1988), and a comparable factor structure (Cole, Hoffman, Tram, & Maxwell, 2000).

The use of the CDI-C and CDI-P in combination may provide a clearer understanding of the child's emotional and behavioral experience. A multi-informant approach is considered a superior way of obtaining information about child psychiatric disorders (Fristad et al., 1991). For instance, parents are superior at reporting child externalizing behaviours and peer relationships while the child is better at reporting their internal experience of emotions and

sleeping patterns (Weissman, Orvaschel, & Padian, 1980).

Cole, D. A., Hoffman, K. B., Tram, J. M., & Maxwell, S. E. (2000). Structural Differences in Parent and Child Reports of Children's Symptoms of Depression and Anxiety. *Psychological Assessment, 12*(2), 174-185.

Cole, D. A., Martin, J. M., Peeke, L. G., Seroczynski, A. D., & Hoffman, K. B. (1998). Are Cognitive Errors of Underestimation Predictive of Reflective of Depressive Symptoms in Children: A Longitudinal Study. *J Abnorm Child Psychol, 107*(3), 481-496.

Fristad, M., Weller, R., Weller, E., Teare, M., & Preskorn, S. (1991). Comparison of the Parent and Child Versions of the Children's Depression Inventory (CDI). *Annals of Clinical Psychiatry, 3*(4), 341-346.

Romano, B. A., & Nelson, R. O. (1988). Discriminant and Concurrent Validity of Measures of Children's Depression. *Journal of Clinical Child Psychology, 17*(3), 255-259.

Weissman, M. M., Orvaschel, H., & Padian, N. (1980). Children's Symptom and Social funcitoning Self-Report Scales. *The Journal of nervous and mental disease, 168*(12), 736-740.

Child Health Questionnaire (CHQ)

The CHQ is from the Child Assessment Project that aimed to develop a reliable measure of children’s physical and psychosocial functioning and wellbeing (Landgraf, Abetz & Ware, 1996). The original 50-item version (CHQ – PF50) has been validated in several countries, including Australia, and population means and psychometric properties for children and adolescent aged 5-18 available (Waters, Salmon, Hesketh & Wake, 2000; Waters, Salmon & Wake, 2000)[3]. The version used in the study is the short-form, which includes the same scales as the CHQ – PF50, but only a subset of the items. This reduces burden and the duration of

completion. Questions are presented in nine sections, such as child’s everyday activities, child’s pain and child’s self-esteem. Questions about the impact of the child’s health status on the parent are also included. Except for three general questions, the questions refer to the 4 weeks preceding the assessment. Response options are on a Likert formats with 4, 5 or 6 options.

The reliability of the short version has been investigated more recently (Raat, Botterweck, Landgraf, Hoogeveen & Essink-Bot, 2005). The physical and psychosocial summary scores showed adequate internal consistency (Cronbach’s alpha > .80), while considering the single scales it ranged between .34 and .85. Test-retest reliability (ICC) was higher for the psychosocial summary measure than the physical (.78 and .44 respectively) while for the single scales it ranged from .14 to .78. The CHQ score correlated with the VAS rating of the parent about how good/bad they judge their child health state and it showed to be able to discriminate between children with a chronic condition such as asthma and frequent headache and children without.

Landgraf, J.M., Abetz, L., Ware, J.A. (1996). *The CHQ user’s manual*. 1st ed. Boston: The Health Institute, New England Medical Centre.

Waters, E., Salmon, L., Hesketh, K., Wake, M. (2000). The Child Health Questionnaire in Australia: reliability, validity and population means. *Aust N Z J Public Health*. 24: 207-10

Waters, E., Salmon, L., Wake, M. (2000). The parent-form child health questionnaire in Australia: comparison of reliability, validity, structure and norms. *J Pediatr Psychol.* 25 (6) 381-391

Raat, H., Botterweck, A.M., Landgraf, J.M., Hoogeveen, W.C., Essink-Bot, M.L. (2005). Reliability and validity of the short form of the child health questionnaire for parents

(CHQ-PF28) in large random school based and general population samples. *J Epidemiol Community Health*. 59:75–82

Children's Report of Parental Behaviour Inventory—Parent Report (CRPBI-PR)

The CRPBI (Schaefer, 1965) was developed on the theoretical assumption that children's perceptions of parental behaviour directed towards them influences their social and personality development. The CRPBI-PR was created through minor changes to the stem and adjustment of the questions of the CRPBI, and the 56-item version was employed (Margolies & Weintraub, 1977). The 56-item version has six subscales (i.e., *Acceptance, Control through Guilt, Nonenforcement, Lax Discipline, Child-centredness,* and *Instilling Persistent Anxiety*), but all versions consistently load on three parenting factors: a) acceptance versus rejection (e.g., "I cheer my child up when he/she is sad"; b) psychological control versus psychological autonomy (e.g., "I say if my child really cared for me, he/she would not do things that make me worry"); and c) firm control versus lax control (e.g., "I let my child get away with a lot of things"). Mothers were asked to respond twice to each statement – once for their own behaviour, and then again for any current partner, on a 3-point scale (i.e., 2-*very true*, 1-*somewhat true,* 0-*not at all true*). A sample of sixth graders, and their mothers and fathers, yielded satisfactory 6-month test-retest reliability (.75 for fathers and .76 for mothers) and good internal consistency (alphas ranging from .91 to .95 for mothers, fathers, and adolescents; Almeida & Galambos, 1991). Interrater agreement and concurrent validity has also been demonstrated (Schwartz et al., 1985). Higher scores reflect greater amounts of acceptance, psychological control, and firmness.

Almeida, D. M., & Galambos, N. L. (1991). Examining father involvement and the quality of father–adolescent relations. Journal of Research on Adolescence, 1, 155–172.

Margolies, P.J., & Weintraub, S. (1977). The revised 56-item CRPBI as a research instrument: reliability and factor structure. J Clin Psychology, 33(2), 472-476.

Schaefer, E. S. (1965). Children's reports of parental behavior: An inventory. Child Development, 36, 413–424.

Schwarz, J. C., Barton-Henry, M. L., & Pruzinsky, T. (1985). Assessing child-rearing behaviors: A comparison of ratings made by mother, father, child and sibling on the CRPBI. Child Development Special Issue: Family Development, 56, 462–479.

<u>The Pubertal Development Scale - Parent Report (PDS-PR)</u>

The PDS-PR is a non-invasive questionnaire measure of pubertal development, and is an adapted version of the scale of Petersen and colleagues (Petersen et al., 1988). The version used is completed by parents, and for female children comprises nine items (including date of onset for menarche, if applicable), and for male children comprises 11 items. The PDS has been demonstrated to be both a valid and reliable measure of pubertal development in children, showing high levels of consistency with other more direct measures of development (Bond et al., 2006; Shirtcliff et al., 2009).

Bond, L., Clements, J., Bertalli, N., Evans-Whipp, T., McMorris, B. J., Patton, G. C., . . . Catalano, R. F. (2006). A comparison of self-reported puberty using the Pubertal Development Scale and the Sexual Maturation Scale in a school-based epidemiologic survey. *J Adolesc, 29*(5), 709-720.

Petersen, A. C., Crockett, L., Richards, M., & Boxer, A. (1988). A self-report measure of pubertal status: Reliability, validity, and initial forms. *Journal of Youth and Adolescence, 17*, 117-133.

Shirtcliff, E. A., Dahl, R. E., & Pollak, S. D. (2009). Pubertal development: correspondence between hormonal and physical development. *Child Dev, 80*(2), 327-337.

The Sexual Maturity Status – Parent Report (SMS-PR)

The SMS-PR is a series of stylised line drawings of girls/boys bodies at differing stages of pubertal development (Morris & Udry, 1980). Parents with a female child are asked to look at a page with five stages of breast development, and five stages of hip and pubic hair development, and asked to circle a number above the two images that most accurately represent their daughter's development. Parents with a male child complete the same task, but with five stages of male genital and pubic hair development in the one set of five images. These images directly correspond to the Tanner stages of pubertal development, and have shown good reliability with physician ratings (Dorn & Biro, 2011).

Dorn, L. D., & Biro, F. M. (2011). Puberty and Its Measurement: A Decade in Review. *Journal of Research on Adolescence, 21*(1), 180-195.

Morris, N. M., & Udry, J. R. (1980). Validation of a Self-Administered Instrument to Assess Stage of Adolescent Development. *Journal of Youth and Adolescence, 9*(3), 271-280.

The Child Behaviour Checklist (CBCL)

The CBCL is a self-report parent/guardian questionnaire aimed at identifying behavioural and emotional problems experience within the past 6 months, for children aged from 4 to 18 years. The first section refers to the child's competence—relative to their peers—across a number of areas including: academic, sporting, social behaviours. The remaining 113

questions ask the parent to identify the presence of emotional and behavioural difficulties. For example 'refuses to talk', 0 = not true, 1 = somewhat true, 2 = very true. Ratings are clustered to form 6 possible categories: anxious/depressed, sleep problems, aggressive behaviour, somatic problems, withdrawn and destructive behaviour. The CBCL has been validated and normed in large community samples (Achenbach & Ruffle, 2000), is a valid indicator of internalizing and externalizing behaviours in children (Schludermann & Schludermann, 1970; Warnick, Bracken, & Kasl, 2008), and has strong reliability and validity (Achenbach & Rescorla, 2001; Sontag-Padilla et al., 2012).

Achenbach, T. M., & Rescorla, L. A. (2001). Manual for the Aseba School-Age Forms & Profiles.

Achenbach, T. M., & Ruffle, T. M. (2000). The Child Behavior Checklist and Related Forms forAssessing Behavioral/Emotional Problems and Competencies. *Pediatrics in review / American Academy of Pediatrics, 21*(8), 265-271.

Schludermann, E., & Schludermann, S. (1970). Replicability of Factors in Children's Report of Parent Behavior (CRPBI). *The Journal of Psychology, 76*(2), 239-249.

Sontag-Padilla, L. M., Dorn, L. D., Tissot, A., Susman, E. J., Beers, S. R., & Rose, S. R. (2012). Executive functioning, cortisol reactivity, and symptoms of psychopathology in girls with premature adrenarche. *Dev Psychopathol, 24*(1), 211-223.

The Lifetime Incidence of Traumatic Events Parent Report (LITE-PR)

The LITE-PR was developed as a screening instrument to assess the type of trauma or loss that a child has experienced (Greenwald & Rubin, 1999). Greenwald also developed a student version of this measure (LITE-S). The LITE-PR includes 16 items that screen for exposure to car accidents, death of family members, parental conflict and other distressing events.

Participants respond yes or no to each event, record how many times it occurred, at what age, how bad the child felt at the time and whether it still bothers them now. The author reports that the measure has good reliability and adequate validity (Greenwald & Rubin, 1999). Although the LITE-PR is a screening measure, for research purposes it has also been used to assess exposure to trauma with various scoring methods used including simply summing the number of events endorsed. It has been used in recent studies reporting significant associations with mental health symptoms (Gustafsson et al., 2009 a,b), including PTSD (Nilsson et al., 2012), and hair cortisol levels in children (Simmons et al, 2016). The 16-item LITE-PR was adapted by removing the two items on sexual abuse and adding items covering mother-child separations and domestic relocation.

Greenwald, R., & Rubin, a. (1999). Assessment of Posttraumatic Symptoms in Children: Development and Preliminary Validation of Parent and Child Scales. *Research on Social Work Practice*, *9*(1), 61–75.

Gustafsson, P.E., Larsson, I., Nelson, N., Gustafsson, P.A., 2009a. Sociocultural disadvantage, traumatic life events, and psychiatric symptoms in preadolescent children. Am. J. Orthopsychiatry 79 (3), 387–397.

Gustafsson, P.E., Nilsson, D., Svedin, C.G., 2009b. Polytraumatization and psychological symptoms in children and adolescents. Eur. Child Adolesc. Psychiatry 18 (5), 274–283.

Nilsson, D.K., Gustafsson, P.E., Svedin, C.G., 2012. Polytraumatization and trauma symptoms in adolescent boys and girls: interpersonal and noninterpersonal events and moderating effects of adverse family circumstances. J. Interpers. Violence 27 (13), 2645–2664.

Simmons, J. G., Badcock, P. B., Whittle, S. L., Byrne, M. L., Mundy, L., Patton, G. C., et al. (2016). The lifetime experience of traumatic events is associated with hair cortisol concentrations in community-based children. *Psychoneuroendocrinology, 63*, 276–281.

The Multidimensional Neglectful Behaviour Scale – Parent Report (MNBS-PR).

The MNBS-PR was developed to estimate the prevalence of neglect and specific sub-types of neglect in community samples (Kantor et al., 2004). The MNBS assesses child neglect across four core domains: physical needs such as food, clothing, shelter, medical care; emotional needs such as affection, companionship, support; supervision needs such as limit setting, attending to misbehaviour, knowing child's whereabouts and friends; and, cognitive needs such as being played with or read to, assisting with school homework (Kantor et al., 2004). The MNBS-PA form was used, for parents of children aged 5-15 years, with the options for younger children used. Parents were not asked the questions related to Abuse, Fighting or Alcohol, as were covered elsewhere. Parents were asked to consider the 39 items over the past six months and respond using the following scale, 1 = 'not at all like you' to 4 = 'really a lot like you'. Adequate levels of reliability and validity have been reported (Kantor et al., 2004). The various verions of the MNBS can be found at: http://pubpages.unh.edu/~mas2/NS7.pdf.

Kaufman Kantor, G., Holt, M., & Straus, M. A. 2004. "The parent-report multidimensional neglectful behavior scale." Durham, NH: Family Research Laboratory.

Child Questionnaires

Attachment Questionnaire for Children (AQC)

The AQC by Muris, Meesters, van Melick and Zwambag (2001) is an abridged version of Hazan and Shaver's (1987) instrument to assess attachment style. Participants are presented with three descriptions of thoughts and perceptions about their friendships with other children. The child is then asked to choose the option that describes them most accurately. Items describe: 1) secure attachment, 'I find it easy to become close friends with other children. I trust them and I am comfortable depending on them. I do not worry about being abandoned or about another child getting too close friends with me'; 2) avoidant attachment, e.g. 'I am uncomfortable to be close friends with other children. I find it difficult to trust them completely, difficult to depend on them. I get nervous when another child wants to become close friends with me. Friends often come more close to me than I want them to', and 3) ambivalent attachment, e.g. 'I often find that other children do not want to get as close as I would like them to be. I am often worried that my best friend doesn't really like me and wants to end our friendship. I prefer to do everything together with my best friend. However, this desire sometimes scares other children away.' By choosing one of these items participants classify their attachment style as secure, avoidant or ambivalent.

Muris, P., Meesters, C., Melick, M. Van, & Zwambag, L. (2001). Self-reported attachment style , attachment quality , and symptoms of anxiety and depression in young adolescents, *30*, 809–818.

Hazan, C., & Shaver, P. (1987). Romantic love conceptualized as an attachment process. *Journal of Personality and Social Psychology, 52*(3), 511–524.

Brief Multidimensional Students Life Satisfaction Scale (BMSLSS) [Phase 2 only]

The BMSLSS (Seligson, Huebner & Valois, 2003; Huebner, Suldo, Valois & Drane, 2006) is an abridged version of the Multidimensional Student's Life Satisfaction Scale (MSLSS: Huebner, 1994). The BMSLSS consists of five items each measuring child and adolescent life satisfaction in one of five domains: family, school, friends, self and living environment. Participants respond using a 7-point Likert scale: 1=terrible, 2=unhappy, 3=mostly dissatisfied, 4=mixed (equally satisfied and dissatisfied), 5=mostly satisfied, 6=pleased, 7=delighted. Participant responses are then summed and divided by number of items resulting in a mean score. Prior research has reported that the measure has good reliability and validity (Heubner, Antaramian, Hills, Lewis & Saha, 2011).

Huebner, E. S. (1994). Preliminary Development and Validation of a Multidimensional Life Satisfaction Scale for Children. *Psychological Assessment*, *6*(2), 149 – 158.

Huebner, E. S., Antaramian, S. P., Hills, K. J., Lewis, A. D., & Saha, R. (2011). Stability and predictive validity of the brief multidimensional students’ life satisfaction scale. *Child Indicators Research*, *4*(1), 161–168.

Huebner, E. S., Suldo, S, M., Valois, R. F., &, & Drane, J. W. (2006). The Brief Multidimensional Students’ Life Satisfaction Scale: Sex, Race, and Grade Effects for Applications with Middle School Students. *Applied Research in Quality of Life*, *1*(2), 211–216.

Seligson, J. L., Huebner, S. E., Valois, R. F. (2003). Preliminary Validation of the Brief Multidimensional Students’ Life Satisfaction Scale (BMSLSS). *Social Indicators Research*, *61*(2), 121–145.

The Children's Depression Inventory 2 (CDI-2)

The CDI-2 is a brief self-report measure of the cognitive, affective and behavioural signs of depression in children and adolescents 7 to 17 years old (Kovaks, 2004). The CDI-2 has two scales (Emotional Problems, Functional Problems) and four subscales (Negative Mood/Physical Symptoms, Negative Self-Esteem, Interpersonal Problems, Ineffectiveness). The CDI-2 has normative data in the relevant age range (Kovaks, 2004), as well solid evidence for reliability and validity (Masip, Amador-Campos, Gómez-Benito, & del Barrio Gándara 2010).

Kovaks, M. (2004). *Children's Depression Inventory*. Toronto, Canada: Multi-Health Systems.

Masip, A. F., Amador-Campos, J. A., Gómez-Benito, J., & del Barrio Gándara, V. (2010). Psychometric properties of children's depression inventory in clinical and community sample. *Spanish Journal of Psychology, 13*(2), 990-999.

The Children's Rejection Sensitivity Questionnaire (CRSQ)

Part one of the CRSQ is a 12-item, self-report measure. Participants imagine themselves in scenarios with a high risk of interpersonal rejection; participant responses indicate their thoughts and feelings while anticipating rejecting or accepting social feedback (Downey, Lebolt, Rincón & Freitas, 1998). For each scenario participants respond to three questions on a six-point likert scale: 'How nervous would you feel right then?' (1=not nervous, 6=very, very nervous), 'How mad would you feel?' (1=not mad, 6=very, very mad), and a third question to gauge whether the participant believes they will be accepted (1=Yes, 6=No). The CRSQ measures children's anxious/angry anticipation of rejection, feelings of dislike/rejection after ambiguous social interactions, and tendency to overact to rejection.

Downey, G., Lebolt, A., Rincón, C., Freitas, A. L., Downey, G., Lebolt, A., … Freitas, A. L. (2016). Rejection Sensitivity and Children's Interpersonal Difficulties. *Child Development, 69*(4), 1074–1091.

Four-Component Empathy Questionnaire for Children (Phase 2 only)

This self-report questionnaire is comprised of the Adolescent Measure of Empathy and Sympathy (AMES; Vossen, Piotrowski, & Valkenburg, 2015) and a section of the Empathic Responsiveness Questionnaire (Olweus & Endresen 1998). Collectively they measure four components of empathy including cognitive empathy (otherwise known as theory of mind), affective sharing, sympathy (otherwise known as empathic concern), and empathic distress.

The AMES contains three subscales, each containing four items, for a total of 12 items, rated on a 5-point Likert scale. Cognitive empathy is defined in the AMES as comprehension or understanding of another person's emotion. An example of an item is "I can often understand how people are feeling even before they tell me". Affective empathy is defined in the AMES as the experience of another person's emotion, and an example item is "When a friend is scared, I feel afraid". Sympathy is defined in the AMES as feeling concern or sorrow for another person's distress, and an example item is "I feel sorry for someone who is treated unfairly". It has been validated in 10 to 15 year olds, and has good psychometrics.

The Empathic Responsiveness Questionnaire has subscales relating to sympathy/empathic concern and empathic distress. Due to the fact that the AMES already covers sympathy/empathic concern, only the Empathic Distress subscale was used. Empathic distress is defined by Olweus and Endresen (1998) as the tendency to experience feelings of discomfort, uneasiness and distress when exposed to the distress of others. The original subscale had four items, but two of these were combined ("When I see a girl who is distressed I sometimes feel like crying" and "When I see a boy…" was turned into "When I see a

girl/boy…") .The three items are ranked on a Likert scale of 1 to 6. The scale has good psychometric properties. This scale has been used in children from 11 years of age, and the language was deemed appropriate for our 9 and 10 year olds. The total questionnaire takes approximately 5 minutes to complete.

Olweus, D., & Endresen, I. M. (1998). The Importance of Sex-of-Stimulus Object: Age Trends and Sex Differences in Empathic Responsiveness. *Social Development, 7*(3), 370-388.

Vossen, H. G. M., Piotrowski, J. T., & Valkenburg, P. M. (2015). Development of the Adolescent Measure of Empathy and Sympathy (AMES). *Personality and Individual Differences, 74*(0), 66-71.

Edinburgh Handedness Inventory (EHI)

The EHI can be administered as an interview, observational assessment or self-report questionnaire to establish the dominant hand (Oldfield, 1971). Items consist of a series of activities (for example, writing or throwing), and participants are asked to indicate their hand preference for each. The EHI is completed in the current study as an observational interview, where children are asked to mimic the activity described. This decreases self-report perceived dominant hand bias. The handedness preference score is used in the analysis and interpretation of MRI data and the examination of laterality effects.

Oldfield, R. C. (1971). The assessment and analysis of handedness: the Edinburgh inventory. *Neuropsychologia, 9*(1), 97-113.

Kerns Security Scale (KSS)

The KSS is a 15-item, forced-choice questionnaire designed to measure attachment in specific attachment relationship, higher scores indicate more secure attachment (Kerns, Klepac & Cole, 1996). The questionnaire assesses the child's relationship to their attachment figure and the child's sense that the attachment figure: 1) is available and responsive (e.g., that they will be there when needed), 2) is someone to rely on when stressed (e.g., tendency to seek attachment figure when upset), 3) is someone to talk to (e.g. tells the parent their thoughts and feelings). Forced choice items are presented e.g., 'Some kids find it easy to trust their mum BUT other kids are not sure if they can trust their mum.' Participants are asked to indicate which response is most like them ('which kids I am like'). They use a four-point likert scale that aligns them with 'some kids' or 'other kids' and then indicate whether this is 'sort of true for me' or 'really true for me' (Harter, 1982).

Kerns, A. K., Klepac, L., & Cole, A. (1996). Peer relationships and preadolescents' perceptions of security in the child-mother relationship. *Developmental Psychology*, *32*(3), 457–466.

Harter, S. (2016). The Perceived Competence Scale for Children. *Child Development*, *53*(1), 87–97.

The Spence Children's Anxiety Scale (SCAS)

The SCAS is a self-report measure that indicates symptoms of anxiety in childhood populations. Made up of 44 items in total, 38 of the items relate to symptoms of anxiety, while the remaining 6 items are of unrelated content to reduce negative bias. Each item is rated on a 4-point scale from "never (0)" "sometimes (1)", "often (2)", "always (3)". The maximum score is 114, and a higher score indicates a greater presence of anxiety symptoms.

The items source a 6-factor model, confirmed by both exploratory and confirmatory factor analyses (Spence, 1998). These factors align with anxiety presentations found in the DSM-IV (Spence, 1997). The six subtypes include; obsessive-compulsive problems, separation anxiety, social phobia, panic, agoraphobia, generalized anxiety symptoms and concerns of physical injury. The SCAS has been identified as a reliable and valid measure across childhood populations with diverse ethnic, cultural and socioeconomic backgrounds (Essau, Muris, & Ederer, 2002; Holly, Little, Pina, & Caterino, 2015; Spence, 1998).
For scoring and further information see http://www.scaswebsite.com/index.php?p=1_7.

Essau, C. A., Muris, P., & Ederer, E. M. (2002). Reliability and validity of the Spence Children's Anxiety Scale and the Screen for Child Anxiety related Emotional Disorders in German children. *Journal of Behavior Therapy and Experimental Psychiatry, 33*, 1-18.

Holly, L. E., Little, M., Pina, A. A., & Caterino, L. C. (2015). Assessment of anxiety symptoms in school children: a cross-sex and ethnic examination. *J Abnorm Child Psychol, 43*(2), 297-309.

Spence, S. H. (1997). Structure of Anxiety Symptoms among Children: A Confirmatory Factor Analytic Study *The American Journal of Psychiatry, 106*(2), 280-297.

Spence, S. H. (1998). A measure of anxiety symptoms among children. *Behavior Research and Therapy, 36*, 545-566.

Teacher Questionnaires

Social Skills Improvement System (SSIS) – Teacher Report

The social skills scale from the SSIS (Gresham, 2008) was used to obtain a measure of the children’s social competence via teacher report. Social skills (both deficits and strengths) in

the following domains are assessed: communication, cooperation, assertion, responsibility, empathy, engagement, and self-control. Teachers indicate the frequency with which the student exhibits each social skill on a 4-point scale of never, seldom, often, and almost always. In addition, teachers indicate the importance of each social skill to the student's development and classroom success using a 3-point scale of not important, important, and critical. Example items are “Makes friends easily”, “Makes a compromise during a conflict” and “Shows concern for others”. The SSIS has been used widely internationally, and has excellent psychometric properties. Specifically, (Crosby, 2011) found that the SSIS teacher measure has good reliability and validity. This scale is completed by the child’s teacher in the form of an online questionnaire, and takes to complete 5 to 10 minutes.

Crosby, J. W. (2011). Test Review: FM Gresham & SN Elliott. *Journal of Psychoeducational Assessment, 29*(3), 292-296.

Gresham, F. M., & Elliott, S. N. (2008). *Social Skills Improvement System: Rating Scales*. Bloomington, MN.
